# Transcriptomic Analysis of the Innate Antiviral Immune Response in Porcine Intestinal Epithelial Cells: Influence of Immunobiotic Lactobacilli

**DOI:** 10.3389/fimmu.2017.00057

**Published:** 2017-02-02

**Authors:** Leonardo Albarracin, Hisakazu Kobayashi, Hikaru Iida, Nana Sato, Tomonori Nochi, Hisashi Aso, Susana Salva, Susana Alvarez, Haruki Kitazawa, Julio Villena

**Affiliations:** ^1^Laboratory of Immunobiotechnology, Reference Centre for Lactobacilli (CERELA-CONICET), Tucuman, Argentina; ^2^Immunobiotics Research Group, Tucuman, Argentina; ^3^Food and Feed Immunology Group, Laboratory of Animal Products Chemistry, Graduate School of Agricultural Science, Tohoku University, Sendai, Japan; ^4^Livestock Immunology Unit, International Education and Research Center for Food Agricultural Immunology (CFAI), Graduate School of Agricultural Science, Tohoku University, Sendai, Japan; ^5^Cell Biology Laboratory, Graduate School of Agricultural Science, Tohoku University, Sendai, Japan; ^6^Infection Immunology Unit, International Education and Research Center for Food Agricultural Immunology (CFAI), Graduate School of Agricultural Science, Tohoku University, Sendai, Japan

**Keywords:** intestinal epithelial cells, immunotranscriptomic response, TLR3, *Lactobacillus rhamnosus* CRL1505, *Lactobacillus plantarum* CRL1506, antiviral response

## Abstract

*Lactobacillus rhamnosus* CRL1505 and *Lactobacillus plantarum* CRL1506 are immunobiotic strains able to increase protection against viral intestinal infections as demonstrated in animal models and humans. To gain insight into the host–immunobiotic interaction, the transcriptomic response of porcine intestinal epithelial (PIE) cells to the challenge with viral molecular associated pattern poly(I:C) and the changes in the transcriptomic profile induced by the immunobiotics strains CRL1505 and CRL1506 were investigated in this work. By using microarray technology and reverse transcription PCR, we obtained a global overview of the immune genes involved in the innate antiviral immune response in PIE cells. Stimulation of PIE cells with poly(I:C) significantly increased the expression of *IFN-*α and *IFN-*β, several interferon-stimulated genes, cytokines, chemokines, adhesion molecules, and genes involved in prostaglandin biosynthesis. It was also determined that lactobacilli differently modulated immune gene expression in poly(I:C)-challenged PIE cells. Most notable changes were found in antiviral factors (*IFN-*α, *IFN-*β, *NPLR3, OAS1, OASL, MX2*, and *RNASEL*) and cytokines/chemokines (*IL-1*β, *IL-6, CCL4, CCL5*, and *CXCL10*) that were significantly increased in lactobacilli-treated PIE cells. Immunobiotics reduced the expression of *IL-15* and *RAE1* genes that mediate poly(I:C) inflammatory damage. In addition, lactobacilli treatments increased the expression *PLA2G4A, PTGES*, and *PTGS2* that are involved in prostaglandin E2 biosynthesis*. L. rhamnosus* CRL1505 and *L. plantarum* CRL1506 showed quantitative and qualitative differences in their capacities to modulate the innate antiviral immune response in PIE cells, which would explain the higher capacity of the CRL1505 strain when compared to CRL1506 to protect against viral infection and inflammatory damage *in vivo*. These results provided valuable information for the deeper understanding of the host–immunobiotic interaction and their effect on antiviral immunity. The comprehensive transcriptomic analyses successfully identified a group of genes (*IFN-*β, *RIG1, RNASEL, MX2, A20, IL27, CXCL5, CCL4, PTGES*, and *PTGER4*), which can be used as prospective biomarkers for the screening of new antiviral immunobiotics in PIE cells and for the development of novel functional food and feeds, which may help to prevent viral infections.

## Introduction

In the past decade, research has demonstrated that beneficial microbes with the capacity to modulate the mucosal immune system (immunobiotics) are a potential alternative to enhance resistance against viral infections. Immunobiotic lactic acid bacteria (LAB) are able to provide protection against viral infections by modulating innate and adaptive antiviral immunity. Several reports have shown that immunobiotic LAB improve protection against enteric viral infections and shorten the duration of diarrhea, reduce the number of episodes, diminish virus shedding, normalize gut permeability, and increase the production of virus-specific antibodies (1–3). Moreover, it was demonstrated that some immunobiotic strains, when orally administered, are able to increase respiratory defenses and reduce the susceptibility to respiratory viral infections improving virus clearance and diminishing inflammatory-mediated lung tissue damage (4–7).

In developing countries, viral mucosal infections such as bronchitis and diarrhea are the most common infectious diseases in children (8–10). The use of immunobiotics to improve the outcome of those viral infections has been proposed. In this regard, in a randomized controlled trial conducted by Villena et al. (4), the immunobiotic strain *Lactobacillus rhamnosus* CRL1505 (administered in a yogurt formulation) improved mucosal immunity and reduced the incidence and severity of intestinal and respiratory infection in children. The incidence of infectious events was reduced from 66% in the placebo group to 34% in the group that received the probiotic yogurt. Furthermore, there was also a significant reduction in the occurrence of indicators of disease severity such as fever and the need for antibiotic treatment in children receiving the probiotic yogurt (4). Studies in mice models have proved that orally administered *L. rhamnosus* CRL1505 improves antiviral immune responses in the intestinal mucosa (local effect) (5, 11) and the respiratory tract (distal effect) (6, 7). Of interest, it was demonstrated that these immunomodulatory capacities are strain specific since other immunobiotic strains such as *Lactobacillus plantarum* CRL1506 exert only local affects after oral administration (5–7, 11).

The interactions of intestinal epithelial cells (IECs) with luminal antigens and immune cells play a central role in determining the type of immune response triggered by microorganisms in the intestinal mucosa (12, 13). Therefore, by using a previously established porcine intestinal epithelial (PIE) cells that is able to respond to the dsRNA synthetic analog poly(I:C) and are permissive to rotavirus (14, 15), we aimed to evaluate the similarities and differences in the innate antiviral immune response induced by *L. rhamnosus* CRL1505 and *L. plantarum* CRL1506. We hypothesized that transcriptomic analyses using microarray technology in PIE cells could provide valuable information to gain insights in the mechanisms involved in the capacity of immunobiotics to modulate the innate antiviral immune response in the gastrointestinal tract and could provide some clues about their ability to stimulate immunity in distal mucosal sites such as the respiratory tract. Therefore, the aim of this study was to investigate the transcriptomic response of PIE cells to the challenge with viral molecular associated pattern poly(I:C) and the changes in that immunotranscriptomic profiles induced by the immunobiotics strains with antiviral capabilities *L. rhamnosus* CRL1505 and *L. plantarum* CRL1506. We obtained a global overview of the immune genes involved in the innate antiviral immune response in PIE cells that include type I interferons (IFNs), several IFN-stimulated genes (ISGs), cytokines, chemokines, adhesion molecules, and genes involved in prostaglandin biosynthesis. It was also determined that lactobacilli differently modulated immune gene expression in poly(I:C)-challenged PIE cells by increasing the expression of antiviral factors and cytokines/chemokines and reducing genes involved in poly(I:C)-mediated inflammatory damage. Moreover, the study allowed us to identify a group of genes that could be used as biomarkers for the screening of new antiviral immunobiotics in PIE cells.

## Materials and Methods

### PIE Cells

PIE cells are intestinal non-transformed cultured cells originally derived from intestinal epithelia isolated from an unsuckled neonatal swine (16). When PIE cells are cultured, they assume a monolayer with a cobblestone and epithelial-like morphology and with close contact between cells (14, 16, 17). PIE cells were maintained in Dulbecco’s modified Eagle’s medium (DMEM) (Invitrogen Corporation, Carlsbad, CA, USA) supplemented with 10% fetal calf serum, 100 U/ml streptomycin, and 100 mg/ml penicillin at 37°C in an atmosphere of 5% CO_2_. PIE cells grow rapidly and are well adapted to culture conditions even without transformation or immortalization (17–19).

### Microorganisms

*Lactobacillus rhamnosus* CRL1505 and *L. plantarum* CRL1506 belong to CERELA Culture Collection and were originally isolated from goat milk (19). These strains were grown in Man-Rogosa-Sharpe broth at 37°C. For immunomodulatory assays, overnight cultures were harvested by centrifugation, washed three times with sterile PBS, counted in a Petroff-Hausser counting chamber, and resuspended in DMEM until use.

### Immunomodulatory Effect of Lactobacilli in PIE Cells

Evaluation of the immunomodulatory activity of *L. rhamnosus* CRL1505 and *L. plantarum* CRL1506 was performed using PIE cells as described previously (19). PIE cells were seeded at 3 × 10^4^ cells per well in 12-well type I collagen-coated plates (Sumitomo Bakelite Co., Tokyo, Japan) and cultured for 3 days. After changing medium, lactobacilli (5 × 10^8^ cells/ml) were added, and 48 h later, each well was washed vigorously with medium at least three times to eliminate all stimulants. Then cells were stimulated with poly(I:C) (60 μg/ml) for 3, 6, 12, or 24 h for reverse transcription (RT)-PCR studies or for 12 h for microarray studies.

### Microarray Analysis

Total RNA was isolated from lactobacilli-treated and control PIE cells using PureLink RNA Mini Kit (Life Technologies Inc., Gaithersburg, MD, USA) and treated with DNase. RNA integrity of all samples were evaluated by Agilent 2100 Bioanalyzer (Agilent Technologies, Santa Clara, CA, USA), using the RNA 6000 Nano Kit (20). Complementary DNA synthesis was performed using 200 ng of RNA. Hybridization with Porcine (V2) Gene Expression Microarray (Agilent Technologies) was performed at Hokkaido System Science Co. Scanning and digitization of Microarray were done by Agilent Technologies Microarray Scanner and Agilent Feature Extraction 10.7.3.1, respectively.

Data normalization and expression analysis were performed using GeneSpring software version 13.1 (Agilent Technologies). Significant genes up and downregulated in test samples [those stimulated with poly(I:C) or lactobacilli plus poly(I:C)] with respect to control samples [without poly(I:C) stimulation] were identified. Genes with significant changes in transcript abundance were selected on the basis of two criteria: a *t*-test *P* value of less than 0.05, which was considered statistically significant, and a cutoff in transcript abundance of at least twofold. Statistical analysis was conducted using the Limma package from BioConductor in R software (version 3.2.5). Results were expressed as log2 scale (log2 ratio). Genes whose expressions were log2 > 1 and *P* < 0.05 were annotated using PANTHER 11.1 (pantherdb.org). Genes were further analyzed according to Gene Ontology (GO) classification. Microarray data were submitted to NCBI-GEO under the accession number GSE93225.

### Quantitative Expression Analysis by Two-Step Real-time Quantitative PCR (qPCR)

Two-step real-time qPCR was performed to characterize the expression of selected genes in PIE cells. Total RNA was isolated from each PIE cell sample using TRIzol reagent (Invitrogen). All cDNAs were synthesized using a Quantitect RT kit (Qiagen, Tokyo, Japan) according to the manufacturer’s recommendations. Real-time qPCR was carried out using a 7300 real-time PCR system (Applied Biosystems, Warrington, UK) and the Platinum SYBR green qPCR SuperMix uracil-DNA glycosylase with 6-carboxyl-X-rhodamine (Invitrogen). The primers used in this study were described before (19, 20). The PCR cycling conditions were 2 min at 50°C, followed by 2 min at 95°C, and then 40 cycles of 15 s at 95°C, 30 s at 60°C, and 30 s at 72°C. The reaction mixtures contained 5 µl of sample cDNA and 15 µl of master mix, which included the sense and antisense primers. According to the minimum information for publication of quantitative real-time PCR experiments guidelines, β-actin was used as a housekeeping gene because of its high stability across porcine various tissues (14, 15, 20). Expression of β-actin was used to normalize cDNA levels for differences in total cDNA levels in the samples. The quality of the RNA in all experiments was checked by Agilent 2100 Bioanalyzer, and all samples were determined to be suitable for the qPCR assay considering values of A260/A280 and A260/A230 over 2.0 and the RIN value over 9.0.

### Statistical Analysis

Statistical analyses were performed using GLM and REG procedures available in the SAS computer program (SAS, 1994). Comparisons between mean values were carried out using one-way ANOVA and Fisher’s least significant difference test. For these analyses, *P* values <0.05 were considered significant.

## Results

### Immunotranscriptomic Changes in PIE Cells after Poly(I:C) Challenge

**Figure 1:**
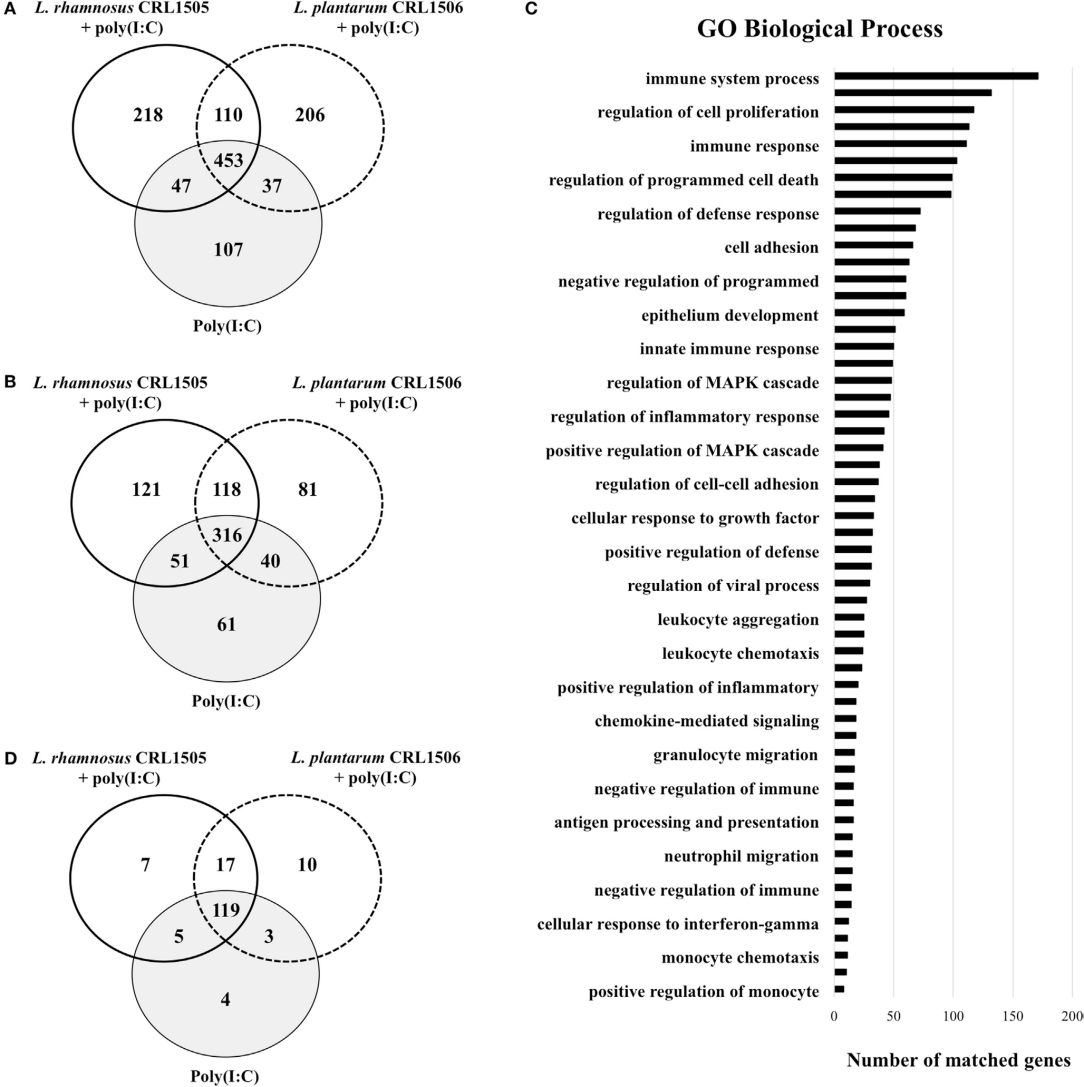
**Differentially regulated genes in porcine intestinal epithelial (PIE) cells treated with immunobiotic *Lactobacillus rhamnosus* CRL1505 or *Lactobacillus plantarum* CRL1506 and challenged with the viral molecular associated pattern poly(I:C)**. Non-lactobacilli-treated PIE cells challenged with poly(I:C) were used as controls. The changes in gene expression were evaluated by comparing the three mentioned groups with unchallenged PIE cells. Venn diagrams showing the number of differentially upregulated **(A)** and downregulated **(B)** genes for each experimental group. Number of matched genes categorized according to Gene Ontology (GO) database **(C)**. Venn diagram showing the number of differentially regulated genes that are known to have immune-related functions for each experimental group **(D)**.

The transcriptomic response of PIE cells to the challenge with poly(I:C) was first investigated. Microarray analysis was performed in PIE cells 12 h after the stimulation with poly(I:C). When these cells were compared with unchallenged PIE cells, it was found that there were 5,140 transcripts (representing 1,178 unique genes) and 3,359 transcripts (representing 788 unique genes) upregulated and downregulated, respectively (Figures 1A,B).

Of these differentially regulated genes, 165 were assigned to immune-related functions according to GO database (Figures 1C,D; Table S1 in Supplementary Material). Changes in the immunotranscriptome response in PIE cells after poly(I:C) stimulation included genes in the following GO Biological Process pathways: “immune system process,” “regulation of defense response,” “cell adhesion,” “innate immune response,” “regulation of viral process,” “cellular response to interferon-gamma,” and several pathways related to immune cells migration and chemotaxis (Figure 1C).

The most remarkable changes in PIE cells after stimulation with poly(I:C) were found in expression type I IFNs and antiviral factors, cytokines, chemokines, adhesion molecules, and prostaglandins.

A significant increase in the expression of *IFN-*β and *IFN-*α was observed in poly(I:C)-challenged PIE cells with fold changes (log_2_ ratio) of 4.3 and 3.5, respectively (Table S1 in Supplementary Material). Increased expression of the IFN-induced antiviral factors *OAS1* (11.2), *OASL* (10.7), *IFIT1* (9.9), *IFIT3* (9.1), *IFIT2* (8.3), *MX1* (7.9), *MX2* (6.3), *OAS2* (6.3), *IFIT5* (3.0), *RNASEL* (2.2), and *RNASE4* (1.9) was also observed. In addition, a significant upregulation of the transcriptional regulators *IRF7* (4.6), *STAT1* (4.3), *IRF1* (3.9), *IRF9* (2.5), and *STAT2* (2.2) were found in poly(I:C)-challenged PIE cells (Table S1 in Supplementary Material).

The stimulation of PIE cells with poly(I:C) significantly increased the expression of the inflammatory cytokines *IL-1*α (4.1), *IL-6* (4.0), and *IL-15* (1.8) (Table S1 in Supplementary Material). There was also a 3.9-fold increase in the expression of the sensor of the inflammasome polymeric complex *NPLR3*. Chemokines involved in monocyte and T lymphocyte recruitment and activation such as *CXCL10* (13.2), *CCL5* (8.4), *CXCL9* (8.1), *CCL4* (7.8), *CCL20* (5.9), *CCL23* (5.1), *CCL28* (2.9), *CCL8* (2.3), and *CCL2* (2.0) were increased after poly(I:C) stimulation. In addition, we observed a significant upregulation of the chemotactic factors for neutrophils *CXCL5* (2.5), *CXCL11* (10.3), and *CXCL8* (1.2) (Table S1 in Supplementary Material). Moreover, *CSF1* (2.9) and *CFS2* (2.9) that are factors able to stimulate the growth and differentiation of hematopoietic precursor cells from granulocytes and macrophages were also increased.

An upregulation of genes for adhesion molecules in PIE cells after stimulation with poly(I:C) was observed, including *SELE* (5.3), *VCAM-1* (4.0), *SELL* (2.6), *ICAM-1* (2.2), *EPCAM* (1.8), and *SELP* (1.8) (Table S1 in Supplementary Material). There was also a sevenfold increase in the expression of *LGALS9* (galectin 9), which is involved in epithelial–lymphocytes interaction.

The microarray analysis revealed increases in the expression of several genes related to prostaglandins biosynthesis in poly(I:C)-challenged PIE cells including *PTGS2* (5.0), *PTGIR* (3.6), *PTGIS* (1.6), *PTGER4* (1.6), and *PLA2G4A* (1.2). In addition, factors belonging to the complement system were upregulated including *C1R* (7.5), *C1S* (5.7), *C3* (2.9), and *CFB* (3.9) (Table S1 in Supplementary Material).

Changes in the expression of some pattern recognition receptors (PRRs) were detected including *TLR2* (1.6), and *PGLYRP2* (8.1). In addition, we detected changes in the viral innate immune receptors *TLR3* (2.8), *DDX58* (8.9) also known as retinoic acid inducible gene-I (*RIG-I*), *IFIH1* (5.4) also known as melanoma differentiation associated gene-5 (*MDA-5*), and *PKR* (3.2) (Table S1 in Supplementary Material). We also detected increases in the expression of serum amyloid A2 (*SAA2*) (8.6).

### qPCR Analysis of Selected Genes in PIE Cells after Poly(I:C) Challenge

To further evaluate gene expression changes induced by poly(I:C) in PIE cells, qPCR was performed. From the 165 immune and immune-related genes differentially regulated by poly(I:C) (Figure 1D; Table S1 in Supplementary Material), we selected 39 belonging to IFN and IFN-induced antiviral factors, cytokines, chemokines, adhesion molecules, prostaglandins, *SAA2, A20, GZMA, LYZ*, and trefoil factor 1 (*TFF1*) to be studied by qPCR. We confirmed that the direction of the changes in gene expression was in agreement with results obtained in the microarray analysis in all the studied genes.

**Figure 2:**
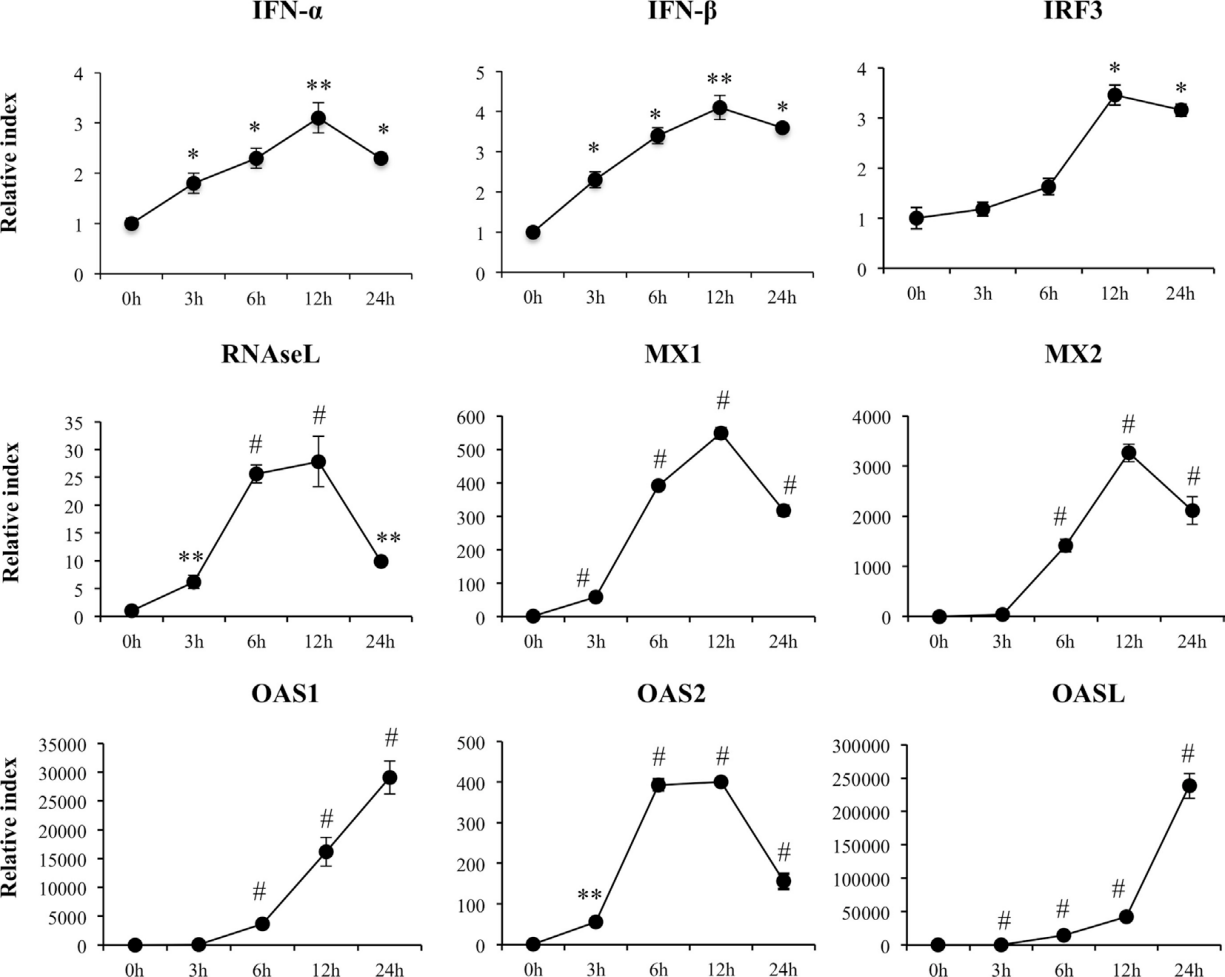
**Expression of type I interferons (*IFN-*β and *IFN-*α), IFN regulatory factor 3, and IFN-induced antiviral genes in porcine intestinal epithelial (PIE) cells after the challenge with the viral molecular associated pattern poly(I:C), analyzed by quantitative PCR**. The results represent data from three independent experiments. Symbols indicate significant differences when compared to unchallenged control PIE cells (time 0 h) (*P < 0.05, **P < 0.01, ^#^*P* < 0.001).

We detected a significant increase in the expression of *IFN-*β and *IFN-*α that reached a maximum value on hour 12 after poly(I:C) stimulation (Figure 2). *IRF3, RNASEL, MX1*, and *MX2* showed a peak on hour 12 after poly(I:C) challenge (Figure 2). Similarly, we observed increases in expression of *OAS1* and *OASL* with peaks at hour 24 and in *OAS2* with peaks between hours 6 and 12 after the poly(I:C) stimulation.

**Figure 3:**
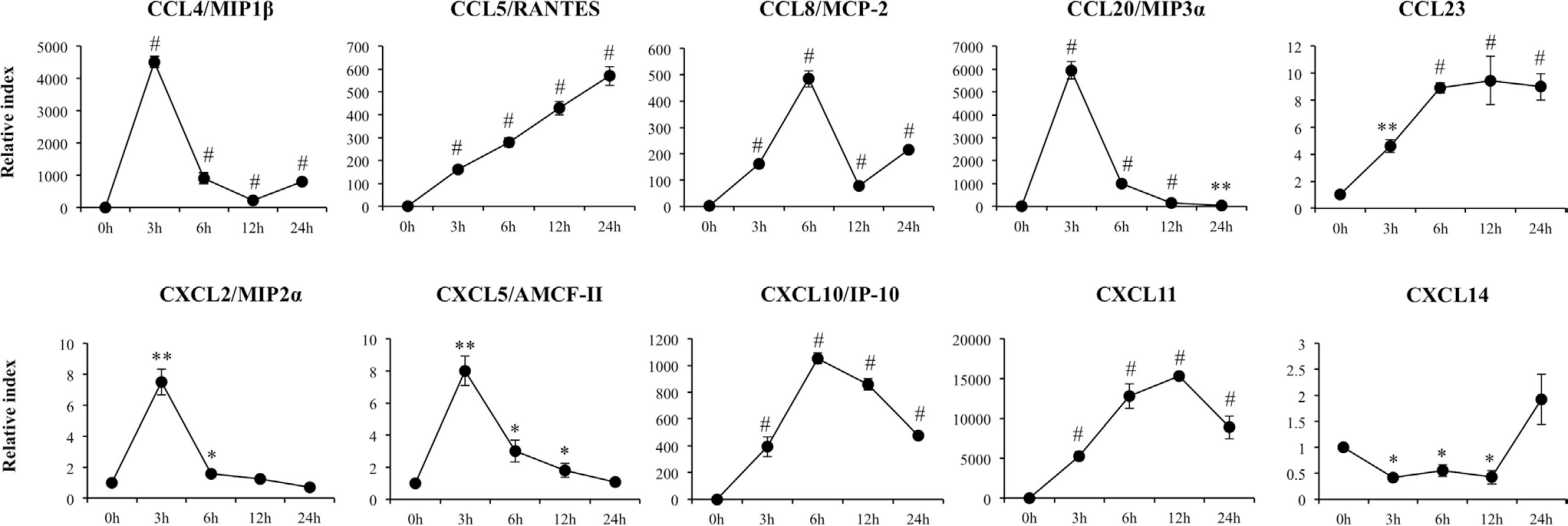
**Expression of chemokines genes in porcine intestinal epithelial (PIE) cells after the challenge with the viral molecular associated pattern poly(I:C), analyzed by quantitative PCR**. The results represent data from three independent experiments. Symbols indicate significant differences when compared to unchallenged control PIE cells (time 0 h) (*P < 0.05, **P < 0.01, ^#^*P* < 0.001).

A significant increase in expression of *CCL4, CCL20, CXCL2*, and *CXCL5* with peaks on hour 3 after poly(I:C) challenge was also detected (Figure 3). Similarly, we observed increases in expression of *CCL8* and *CXCL10* with peaks at hour 6 and in *CCL11* and *CCL5* with peaks at hours 12 and 24, respectively. In addition, expression of *CCL23* increased from hour 3 and stayed in the same level between hours 6 and 24 after stimulation of PIE cells (Figure 3). *CXCL14* expression was significantly reduced after poly(I:C) challenge and returned to basal levels at hour 24. Poly(I:C) also increased the expression of the adhesion molecules *SELE, SELL, ICAM-1*, and *EPCAM* (Figure S1 in Supplementary Material).

**Figure 4:**
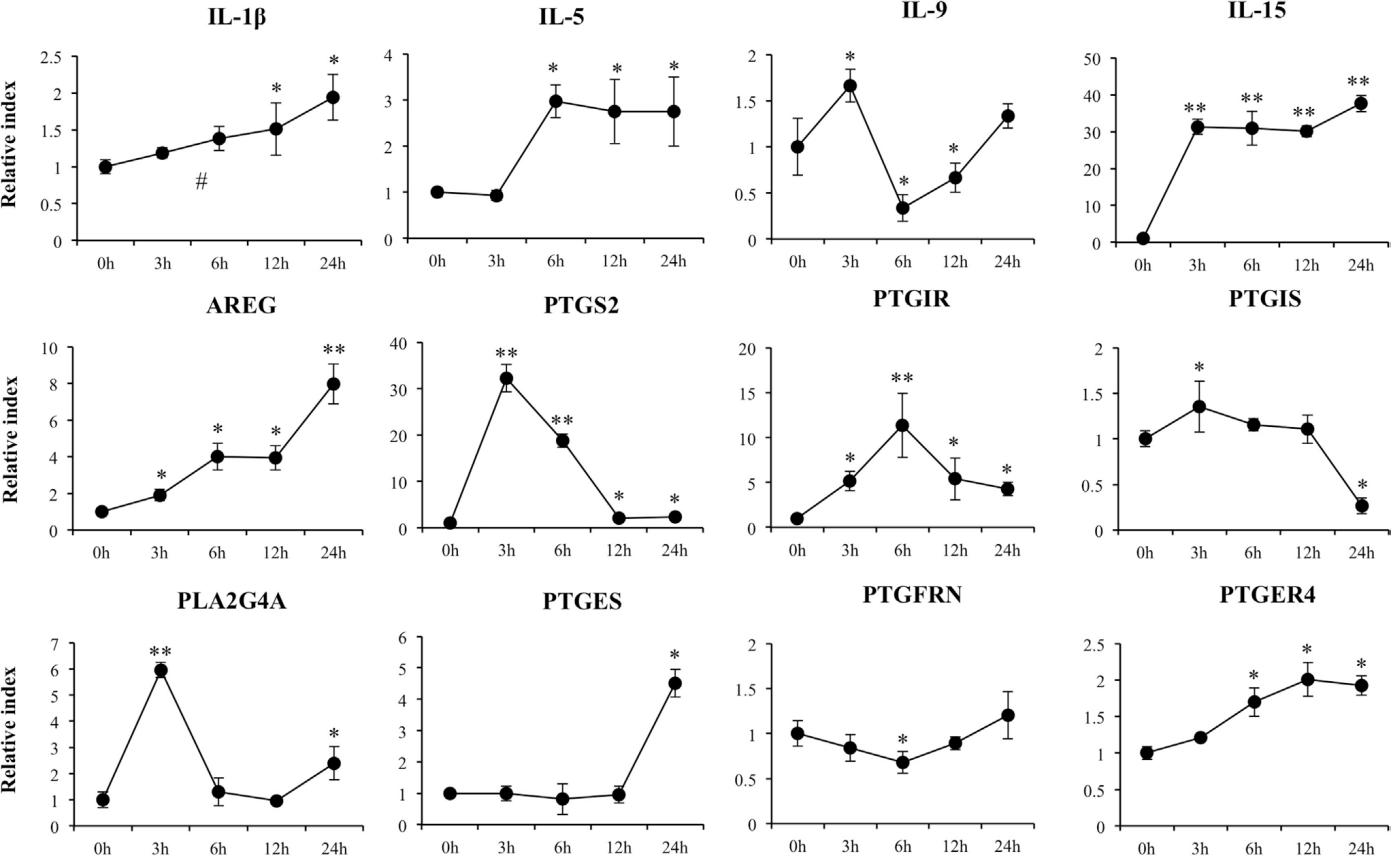
**Expression of cytokines and genes involved in prostaglandins biosynthesis in porcine intestinal epithelial (PIE) cells after the challenge with the viral molecular associated pattern poly(I:C), analyzed by quantitative PCR**. The results represent data from three independent experiments. Symbols indicate significant differences when compared to unchallenged control PIE cells (time 0 h) (*P < 0.05, **P < 0.01, ^#^*P* < 0.001).

Increased expression of *IL-1*β, *IL-5*, and *IL-15* was observed in poly(I:C)-challenged PIE cells (Figure 4) showing all of them their highest values after 6 h of stimulation. Amphiregulin (*AREG*) was also increased after poly(I:C) challenge with a peak at hour 24. On the contrary, *IL-9* expression was significantly reduced between hours 6 and 12 and returned to the basal levels at hour 24 (Figure 4).

We detected a significant increase in the expression of *PTGS2, PTGIR, PLA2G4A, PTGES*, and *PTGER4* (Figure 4). In addition, a slight increase in *PTGIS* was observed at hour 3, and a significant downregulation occurred at hour 24. A decrease in *PTGFRN* between hours 3 and 12 was also observed (Figure 4).

Finally, we observed upregulation of *GZMA, LYZ, TFF1*, and *SAA2* with peaks at hours 3, 6, 12, and 24, respectively (Figure S2 in Supplementary Material).

### Modulation of Poly(I:C)-Induced Immunotranscriptome Changes in PIE Cells by Immunobiotics

Next, we analyzed microarray data to evaluate the effect of the immunobiotic strains *L. rhamnosus* CRL1505 and *L. plantarum* CRL1506 on the immunotranscriptomic response of PIE cells after the challenge with poly(I:C). For that purpose, PIE cells were stimulated with *L. rhamnosus* CRL1505 or *L. plantarum* CRL1506 and then challenged with poly(I:C). Comparative analysis of microarray profiles indicated that both CRL1505 and CRL1506 strains differentially modulated the expression of several genes related to the innate antiviral immune response in PIE cells after poly(I:C) stimulation (Table S2 in Supplementary Material).

The Venn diagram analysis was used to find genes that were uniquely and commonly modulated between lactobacilli-treated and control PIE cells (Figure 1D). Of the 165 differentially expressed genes in the Venn diagram analysis, 4 (*PPARA, TFF1, STAT3*, and *DUOX1*) were unique to the poly(I:C) challenge. Seven (*TNFRSF11B, C5, LOC100127164, PIK3R5, IL27, IL17RC*, and *IBSP*) and 10 (*NFIA, CADM4, CDH24, CCR7, IL1RAPL2, TNFAIP8L2, DPEP1, CDH19, BPIFA1*, and *TNFSF18*) unique genes were found in the CRL1505 stimulation plus poly(I:C) challenge and the CRL1506 stimulation plus poly(I:C) challenge groups, respectively. In addition, five genes (*RNASE6, PROC, VTN, CCL28*, and *PLG*) were common to CRL1505 treatment plus poly(I:C) and control, whereas three genes (*IL23RA, ITGA1*, and *IL20RB*) were common to CRL1506 treatment plus poly(I:C) and control. It was also observed that 119 genes were common to all the 3 treatments (Figure 1D). The cluster analysis in Figure S3 in Supplementary Material depicts the transcriptomic patterns of differentially modulated genes between lactobacilli-treated and control PIE cells. The treatment with CRL1505 plus poly(I:C) clustered closer to the treatment with CRL1506 plus poly(I:C) and both clustered separated from the control.

Closer examination of gene expression revealed differences in several genes sheared by immunobiotic-treated PIE cells and controls (Table S1 in Supplementary Material). Most remarkable differences were found in the genes belonging to IFN and IFN-induced antiviral factors, cytokines, chemokines, and adhesion molecules. Both lactobacilli treatment significantly increased *IFN-*β, *IFN-*α, *TLR3, OAS1, OASL, MX2, RNASEL, RNASE4*, and *STAT5A* when compared to controls. In addition, stimulation of PIE cells with *L. rhamnosus* CRL1505 plus poly(I:C) significantly increased the expression levels of *IFIT1, IFITM1, DDX58/RIG1, IFIH1/MDA5, IRF7, STAT1, NLRP3, IRF1, STAT2*, and *IRF2* when compared with PIE cells stimulated only with poly(I:C).

Although expression of *IL1A, IL6, IL8, AREG, CXCL10, CCL5, CCL4, CCL20, CCL23, CSF2, CCL3L1*, and *SELL* was upregulated in lactobacilli-treated PIE cells after the challenge with poly(I:C), the increases were significantly higher when compared to control PIE cells without lactobacilli treatment (Table S1 in Supplementary Material). *L. rhamnosus* CRL1505 plus poly(I:C) also increased the expression levels of *VEGFA, IL17RC, CXCL11, CCRL2, CXCL5, CXCL2, SELE, CDHR4*, and *EPCAM* when compared with PIE cells stimulated only with poly(I:C), an effect that was not observed with CRL1506 treatment. Interestingly, *IL27* was upregulated only in PIE cells receiving the CRL1505 strain plus poly(I:C). In addition, the expression levels of *IL15* and *RAE1* were reduced by lactobacilli treatments.

We also observed an increased expression of *PLA2G4A, PTGES*, and *PTGS2* genes in lactobacilli-treated PIE cells after the challenge with poly(I:C) when compared to the control cells, whereas *PTGER4* and *PTGER2* were diminished in lactobacillus-treated cells (Table S1 in Supplementary Material). *L. rhamnosus* CRL1505 plus poly(I:C) also increased the expression levels of *PTGIR*.

Expression of *TLR6, MYD88, NCOA1*, and *NFKB1* was significantly higher in lactobacilli-treated PIE cells after the challenge with poly(I:C) when compared to controls. In addition, the transcripts of other immune and immune-related genes including *GZMH, TFF1, LYZ, C1R, CFB, PLG, CFD, SAA2*, and *NOS2* were higher in lactobacilli-treated PIE cells than controls (Table S1 in Supplementary Material). Stimulation of PIE cells with *L. rhamnosus* CRL1505 plus poly(I:C) significantly increased the expression levels of *C1S, C3*, and *PLAU* when compared with PIE cells stimulated only with poly(I:C).

### qPCR Analysis of Selected Genes in PIE Cells after Stimulation with Immunobiotics and Poly(I:C) Challenge

To confirm the changes induced by *L. rhamnosus* CRL1505 and *L. plantarum* CRL1506 in the immunotranscriptome response of poly(I:C)-challenged PIE cells, qPCR was performed on selected genes. Genes with or without significant differences between lactobacilli-treated and non-treated PIE cells were chosen. The transcriptional changes evaluated by qPCR indicated a similar overall trend in the transcription.

**Figure 5:**
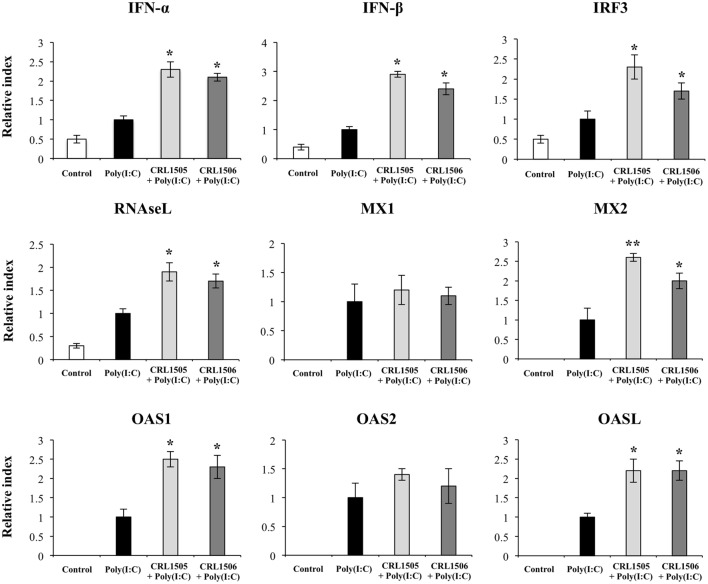
**Expression of type I interferons (*IFN-*β and *IFN-*α), IFN regulatory factor 3, and IFN-induced antiviral genes in porcine intestinal epithelial (PIE) cells treated with immunobiotic *Lactobacillus rhamnosus* CRL1505 or *Lactobacillus plantarum* CRL1506 and challenged with the viral molecular associated pattern poly(I:C), analyzed by quantitative PCR**. Non-lactobacilli-treated PIE cells with or without poly(I:C) challenge were used as controls. The results represent data from three independent experiments. Asterisks indicate significant differences when compared to poly(I:C)-challenged control PIE cells (*P < 0.05, **P < 0.01).

Both *L. rhamnosus* CRL1505 and *L. plantarum* CRL1506 induced a significantly higher expression of *IFN-*α and *IFN-*β when compared with control poly(I:C)-challenged PIE cells (Figure 5). In addition, *IRF3* and the IFN-induced antiviral factors *RNASEL, MX2, OAS1*, and *OASL* were significantly higher in lactobacilli-treated PIE cells than in controls. Furthermore, *MX2* expression was higher in PIE cells treated with CRL1505 strain than those treated with CRL1506. Expression of *MX1* and *OAS2* in lactobacilli-treated PIE cells was not different from the control PIE cells after the challenge with poly(I:C) (Figure 5).

**Figure 6:**
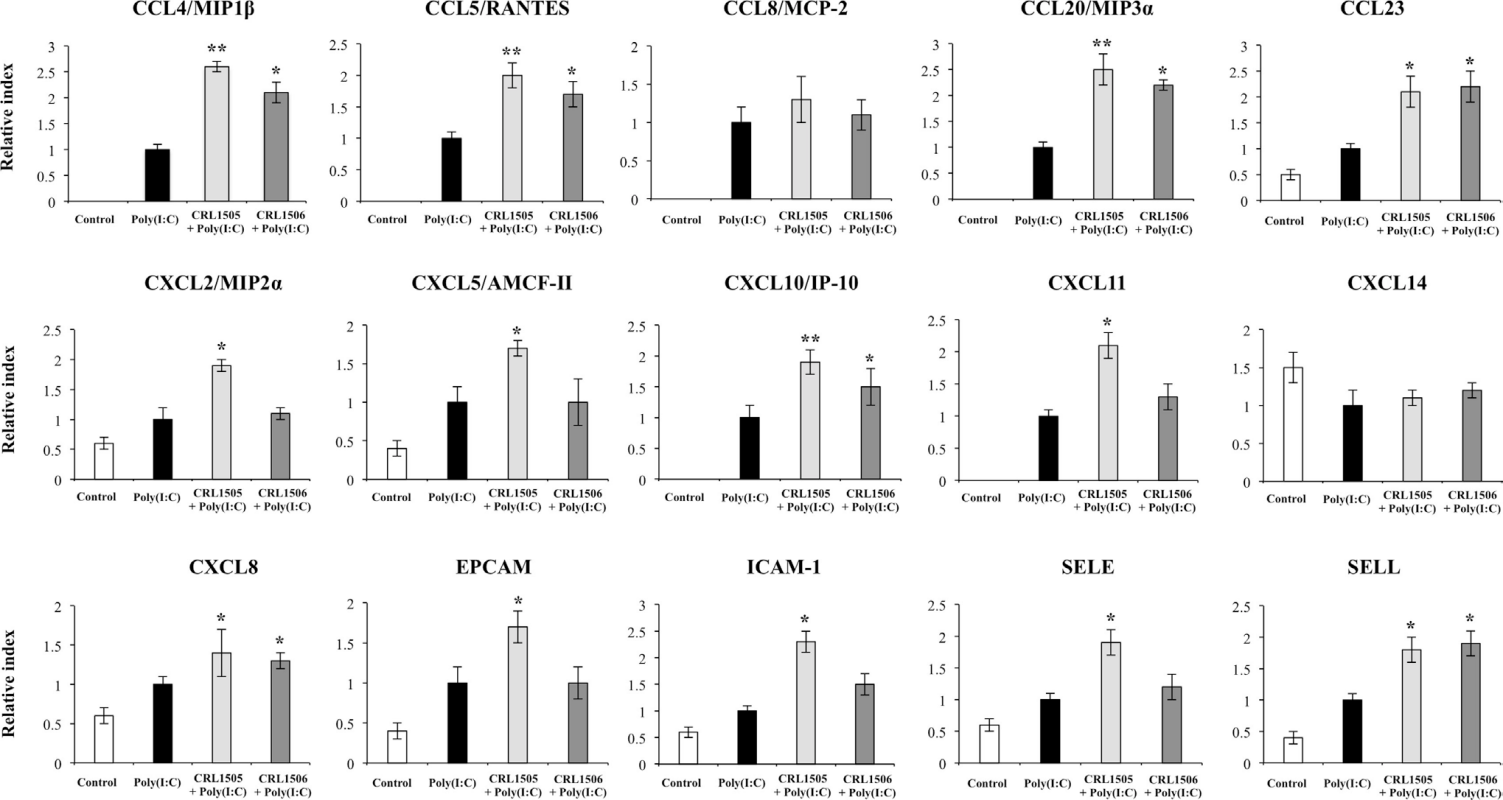
**Expression of chemokines and adhesion molecules genes in porcine intestinal epithelial (PIE) cells treated with immunobiotic *Lactobacillus rhamnosus* CRL1505 or *Lactobacillus plantarum* CRL1506 and challenged with the viral molecular associated pattern poly(I:C), analyzed by quantitative PCR**. Non-lactobacilli-treated PIE cells with or without poly(I:C) challenge were used as controls. The results represent data from three independent experiments. Asterisks indicate significant differences when compared to poly(I:C)-challenged control PIE cells (*P < 0.05, **P < 0.01).

Expression of *CCL8* and *CXCL14* in lactobacilli-treated PIE cells was not different from the control PIE cells after the challenge with poly(I:C) (Figure 6). In contrast, the levels of *CCL23, CXCL8*, and *SELL* were significantly higher in lactobacilli-treated PIE cells when compared to the controls (Figure 6). In addition, both lactobacilli significantly increased the expression of *CCL4, CCL5, CCL20*, and *CXCL10*; however, values in *L. rhamnosus* CRL1505-treated PIE cells were higher than in cells treated with *L. plantarum* CRL1506. Only *L. rhamnosus* CRL1505 was able to increase the expression of *CXCL2, CXCL5, CXCL11, EPCAM, ICAM-1*, and *SELE* when compared to control PIE cells (Figure 6).

**Figure 7:**
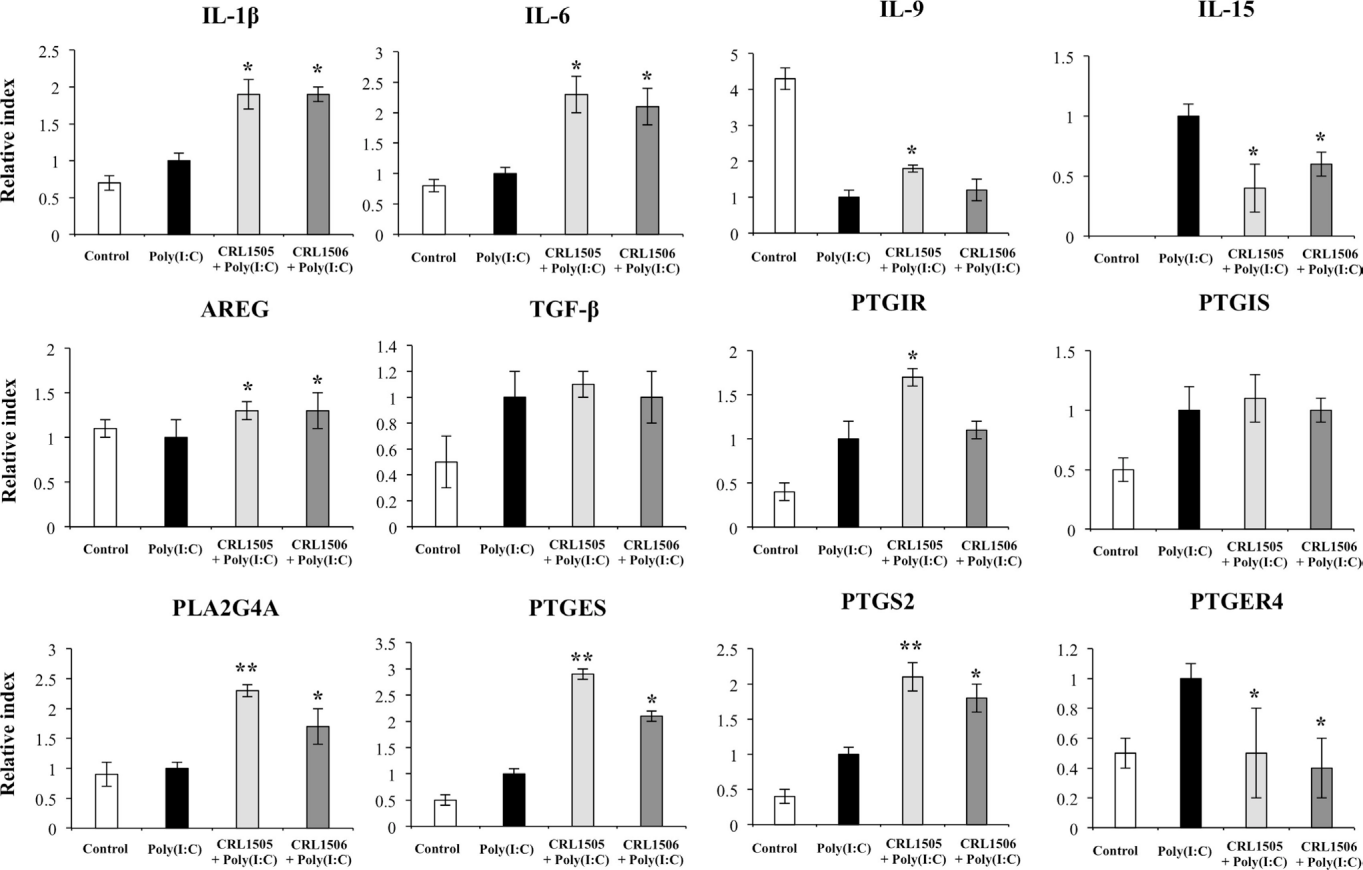
**Expression of cytokines and genes involved in prostaglandins biosynthesis in porcine intestinal epithelial (PIE) cells treated with immunobiotic *Lactobacillus rhamnosus* CRL1505 or *Lactobacillus plantarum* CRL1506 and challenged with the viral molecular associated pattern poly(I:C), analyzed by quantitative PCR**. Non-lactobacilli-treated PIE cells with or without poly(I:C) challenge were used as controls. The results represent data from three independent experiments. Asterisks indicate significant differences when compared to poly(I:C)-challenged control PIE cells (*P < 0.05, **P < 0.01).

In agreement with the results from our microarray analysis, both lactobacilli strains were able to increase the expression of *IL-1*β, *IL-6*, and *AREG* and reduce the expression of *IL-15* and *PTGER4*, with no significant differences between them (Figure 7). Moreover, no differences in *TGF-*β or *PTGIS* were found between lactobacilli-treated and control PIE cells. Both lactobacilli significantly increased the expression of *PLA2G4A, PTGES*, and *PTGS2*; however, values in *L. rhamnosus* CRL1505-treated PIE cells were higher than in cells treated with *L. plantarum* CRL1506. In addition, only *L. rhamnosus* CRL1505 was able to significantly increase the expression of *IL-9* and *PTGIR* when compared to control PIE cells (Figure 7).

**Figure 8:**
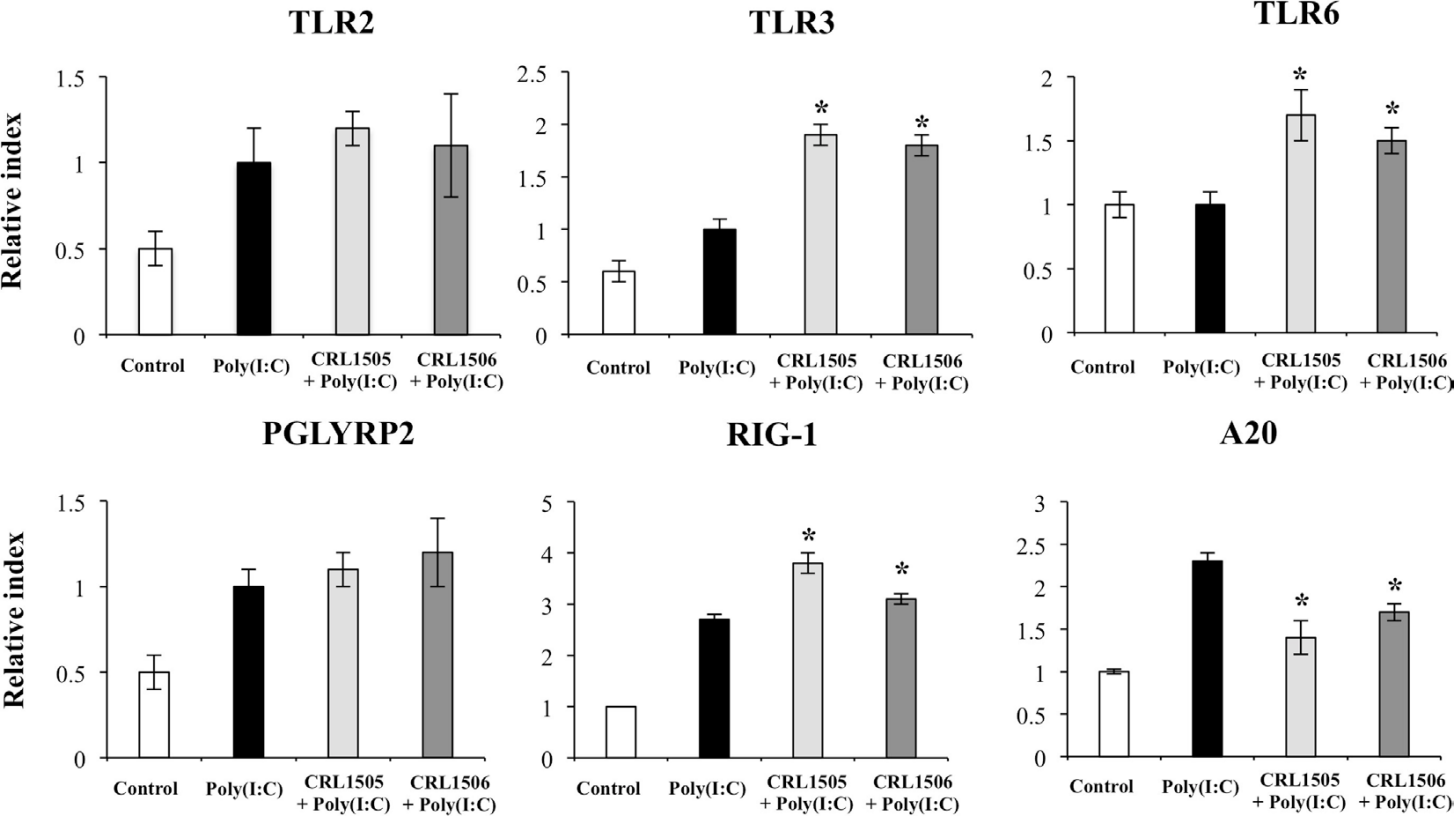
**Expression of pattern recognition receptors and A20 genes in porcine intestinal epithelial (PIE) cells treated with immunobiotic *Lactobacillus rhamnosus* CRL1505 or *Lactobacillus plantarum* CRL1506 and challenged with the viral molecular associated pattern poly(I:C), analyzed by quantitative PCR**. Non-lactobacilli-treated PIE cells with or without poly(I:C) challenge were used as controls. The results represent data from three independent experiments. Asterisks indicate significant differences when compared to poly(I:C)-challenged control PIE cells (*P < 0.05).

Expression of *TLR2* and *PGLYRP2* in CRL1505- or CRL1506-tretaed PIE cells was not different from the control PIE cells after the challenge with poly(I:C). In contrast, expression levels of *RIG1, TLR3*, and *TLR6* (Figure 8) were significantly higher in lactobacilli-treated PIE cells when compared to the controls. We also observed that *A20* (*TNFAIP3*) was reduced in lactobacilli-treated PIE cells when compared to the controls (Figure 8). *SAA2, GZMA, LYZ, TFF1*, and *C1R* were significantly upregulated in lactobacilli-treated PIE cells when compared to the controls (Figure S4 in Supplementary Material). Only *L. rhamnosus* CRL1505 was able to significantly increase the expression of *C3* when compared to control PIE cells, whereas both lactobacilli reduced the expression of *CFB* (Figure S4 in Supplementary Material).

## Discussion

It is known that IECs senses viral dsRNA through PRRs including TLR3, RIG-I, and MDA-5. After the recognition of dsRNA by those receptors, cellular signaling cascades are activated to react against viral infection. Antiviral PRRs activation leads to the production of cytokines, chemokines, IFNs, and IFN-regulated gene products that play a key role in establishing an antiviral state for virus clearance and restriction of spread (21).

High-throughput microarray technology has been employed for screening genes involved in the immune responses to enteric virus or poly(I:C) (22, 23). By using a human colon epithelial cell line (HT29 cells), Bagchi et al. (22) evaluated the immunotranscriptomic response of IECs to the challenge with different rotavirus strains. Microarray data revealed a set of commonly differentially regulated genes for the three rotaviruses used in that work. Of interest, several IFN inducible genes (*OAS1, MX1, IL18, IITP3, TAP1*, and *RSAD2*) as well as several cytokines and chemokines (*CCL5, CXCL10, CXCL11, IL8*, and *CCL15*) were upregulated by rotavirus infection. Later, it was observed that the stimulation of HT29 cells with poly(I:C) enhanced the expression of several genes associated with the dsRNA recognition by PRRs including antiviral factors (*IRF1, ISG20, IFIT2, OASL*, and *STAT5*), and proinflammatory cytokines (*CSF1, CSF2, IL29, TNF-*α, *CXCL11*, and *CLCF1*) (23). Those transcriptomic studies indicated that poly(I:C) and rotavirus induce similar innate antiviral immunotranscriptomic responses in IECs.

Previously, the response of PIE cells to poly(I:C) challenge was evaluated, and it was found that MCP-1, IL-8, TNF-α, IL-6, and both IFN-α and IFN-β were upregulated in PIE cells after stimulation (14). The suitability of PIE cells as a model for studying immune signaling pathways after rotavirus infection was also evaluated. Our results showed that PIE cells have functional TLR3, RIG-I, and MDA-5 receptors, which are able to detect rotavirus infection and enhance the expression of IFN-β and the ISGs MxA and RNase L (15), which are important antiviral effectors of IFN pathway. In this study, we corroborated and deepen those findings by using microarray technology and qPCR. We demonstrated that stimulation with poly(I:C) significantly alters gene expression profiles of PIE cells. Of the transcripts differentially modulated by poly(I:C), several were assigned to immune-related functions. Our results showed that the activation of IRF3 and NF-kB pathways in PIE cells by poly(I:C) increased the expression of IFN-α and IFN-β, several ISGs (*OAS1, OASL, IFIT1, IFIT3, IFIT2, MX1, MX2, OAS2, IFIT5, RNASEL*, and *RNASE4*), cytokines (*IL-1*β, *IL-5*, and *IL-15*), and chemokines (*CCL4, CCL20, CXCL2, CXCL5, CCL8, CXCL10, CCL11, CCL5*, and *CCL23*). Moreover, some adhesion molecules were also significantly upregulated in PIE cells after poly(I:C) stimulation including *SELE, SELL, ICAM-1*, and *EPCAM*. In addition, we also observed a significant upregulation of the dsRNA detection sensors *TLR3, RIG1*, and *MDA5*. This is in agreement with studies in HT29 cells showing that RIG1 was upregulated by rotavirus infection (22).

**Figure 9:**
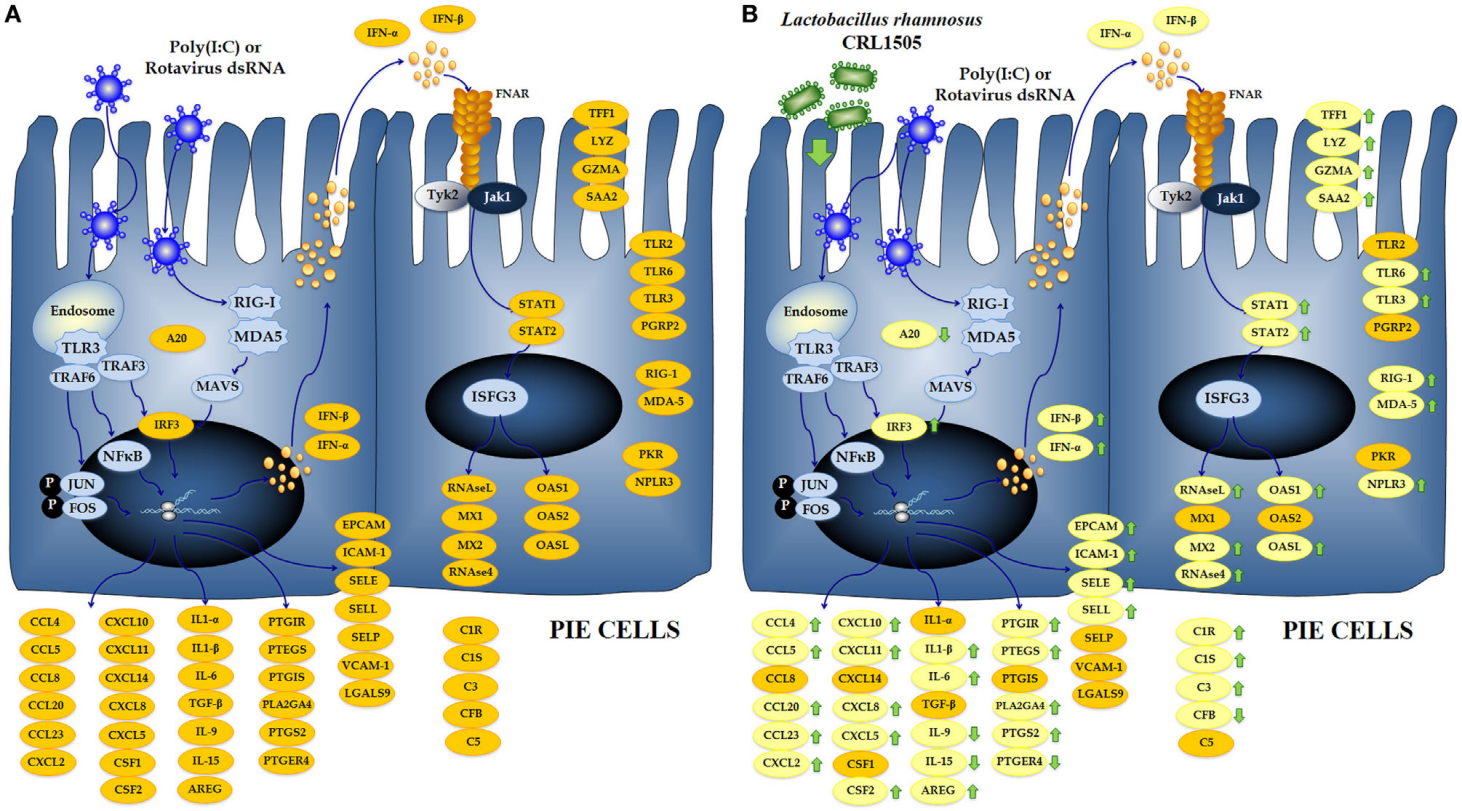
**Global overview of the signaling pathways and immune genes differentially regulated in porcine intestinal epithelial (PIE) cells after the challenge with the viral molecular associated pattern poly(I:C) (A)**. Global overview of the signaling pathways and immune genes differentially regulated in PIE cells treated with *Lactobacillus rhamnosus* CRL1505 and challenged with poly(I:C) **(B)**.

These results are in line with the transcriptomics studies mentioned before and indicate that PIE cells are able to mount a complex innate antiviral immune response involving changes needed to induce a mucosal antiviral state and promote the recruitment of inflammatory cells to the intestinal tissue, which are intended to eliminate the viral pathogen (Figure 9A). These features also exhibit that PIE cells are an excellent laboratory tool to study treatments able to favorably modulate the innate antiviral response.

Several studies have shown that immunobiotics are able to beneficially modulate PRRs-mediated inflammatory response in the gut by regulating the functions of IECs (24). In this regard, our previous studies demonstrated that the immunobiotic strains *L. rhamnosus* CRL1505 and *L. plantarum* CRL1506 differentially regulated the expression of IFN-α, IFN-β, MCP-1, IL-8, and IL-6 in PIE cells after TLR3 activation (19). In line with those previous findings, we described here that the treatment of PIE cells with lactobacilli resulted in differential expression of several immune genes in response to the poly(I:C) challenge (Figure 9B), which include not only antiviral factors and cytokines as previously demonstrated but also adhesion molecules, complement factors, enzymes involved in prostaglandin biosynthesis, and PRRs. Most notable changes were found in *IFN-*α, *IFN-*β, *NPLR3, OAS1, OASL, MX2, RNASEL*, and *RNASE4* that were significantly increased in lactobacilli-treated PIE cells when compared to the controls. It is known that RNAse L, OAS, MX, and NPLR3 are important factors for the protection of the intestinal mucosa against rotavirus infection (25–27). This finding is of interest because it confirms our previous *in vitro* (19) and *in vivo* (11) studies demonstrating the antiviral capacity of *L. rhamnosus* CRL1505 and *L. plantarum* CRL1506.

In addition, it was observed that *L. rhamnosus* CRL1505 and *L. plantarum* CRL1506 differentially regulated the expression of cytokines, chemokines, and adhesion molecules (Figure 9B). Expression levels of *IL-1*β, *IL-6, SELL, CCL4, CCL5, CCL20, CCL23, CXCL8*, and *CXCL10* were higher in lactobacilli-treated PIE cells than controls. In addition, *ICAM1, EPCAM, CXCL2, CXCL5*, and *CXCL11* were increased in CRL1505-tretated PIE cells. This pattern of cytokines/chemokines and adhesion molecules gene expression induced by lactobacilli would allow us to predict an improved recruitment and activation of immune cells to the gut mucosa, which could beneficially influence the elimination of the virus. It is also necessary to consider that in several viral infections, the excessive recruitment of inflammatory cells and/or their deregulated activation may contribute to the damage of the infected tissue rather than the resolution of the infection. It was reported that poly(I:C), when administered intraperitoneally to mice, mimics the local intestinal immune response elicited by an enteric viral infection (28, 29). Both purified dsRNA from rotavirus and poly(I:C) are able to induce severe mucosal damage in the gut *via* TLR3 activation including villous atrophy, mucosal erosion, and gut wall attenuation (28). It was demonstrated that TLR3 activation in IECs by poly(I:C) or rotavirus genomic dsRNA induce the expression of IL-15 and retinoic acid early inducible-1 (RAE1), which mediate epithelial destruction and mucosal injury by interacting with the NKG2D receptor expressed on CD3^+^NK1.1^+^CD8αα^+^ intraepithelial lymphocytes (IELs) (30). Here, we found a significant reduction in the expression of *IL-15* and *RAE1* in PIE cells treated with lactobacilli. This is in line with our previous work that showed that mice pretreated with immunobiotic lactobacilli responded with reduced levels of TNF-α, IL-15, RAE1, and CD3^+^NK1.1^+^CD8αα^+^ IELs after TLR3 activation with poly(I:C) (11). Those changes significantly diminished the inflammatory damage of the intestinal mucosa.

Our transcriptomic study indicates that other regulatory mechanisms would be improved by lactobacilli to limit the inflammatory damage during intestinal viral infection. A significant upregulation of *AREG* and *TFF1* expression was observed in lactobacilli-treated PIE cells when compared to controls. Recently, it was demonstrated that the mucosal surfaces of lung and intestine are protected from detrimental inflammation by group 2 innate lymphoid cells (ILC2s). Monticelli et al. (31) showed that following activation with IL-33, ILC2s in the gut increased the expression of *AREG*, limited intestinal inflammation, and decreased disease severity in mice treated with dextran sodium sulfate. Moreover, it was reported that the number of ILC2s increased in the respiratory tract after infection influenza virus and that depletion of those cells induced impaired airway remodeling and altered lung epithelial integrity, diminishing lung function. Notably, these defects were restored by administration of AREG (32). On the other hand, TFF1 is a stable secretory protein expressed in gastrointestinal mucosa that stabilize the mucus layer and affect healing of the epithelium. By using TFF1-knockout mice, it was showed that this factor plays a critical role in maintaining mucosal integrity and regulating the pro-inflammatory response to gastrointestinal pathogens (33). Moreover, a recombinant *Lactococcus lactis* strain, genetically modified to secrete human TFF1, was able to reduce the severity of mucosal damage in an animal model of oral mucositis (34).

We also observed that poly(I:C) stimulation induced transcriptomic changes in several genes involved in the biosynthesis of prostaglandins and that lactobacilli treatments increased the expression of several of those genes including *PLA2G4A, PTGES*, and *PTGS2*. Upregulation of *PLA2G4A* and *PTGES* indicates that PIE cells treated with lactobacilli would increase their production of prostaglandin E2 (PGE2). It has been reported that PGE2 regulates immune function in several ways that are able to affect viral pathogenesis. Production of pro-inflammatory cytokines and chemokines by immune cells are inhibited in presence of PGE2, whereas IL-10 is enhanced (35, 36) indicating that PGE2 could have a role in the protective activity of *L. rhamnosus* CRL1505 and *L. plantarum* CRL1506 against inflammatory damage. It was also reported that PGE2 inhibits type I IFN production in epithelial and immune cells, thereby causing an increase in virus replication (37). Interestingly, the expression of PGE2 receptors (*PTGER4* and *PTGER2*) was downregulated in PIE cells treated with the immunobiotic strains indicating that cells were protected from this effect of PGE2.

Whether the capacity of *L. rhamnosus* CRL1505 and *L. plantarum* CRL1506 to differentially modulate AREG, TFF1, and prostaglandins production is involved in their beneficial effects on intestinal or respiratory viral infections *in vivo* is an open question, which we propose to address in the near future.

The zinc-finger protein A20 is capable to terminate TLR signaling, which results in inhibition of NF-κB activation and reduction of inflammatory-induced cytotoxicity (38). Saitoh et al. (39) reported that IRF3 activation is suppressed by A20. The A20 protein is able to induce the suppression of the IFN-mediated immune response and IFN-promoter-dependent transcription following engagement of TLR3 by dsRNA. A20 knock down results in enhanced IRF3-dependent transcription triggered by the stimulation of TLR3 or virus infection. Furthermore, it was reported that A20 was upregulated by different rotavirus strains in HT29 cells. Interestingly, the same work demonstrated that the knock down of A20 in IECs by siRNA significantly reduced virus titers indicating that A20 is required for rotavirus infection (39). We have reported previously that two immunobiotic bacteria with antiviral capabilities, *Bifidobacterium infantis* MCC12 and *Bifidobacterium breve* MCC1274, significantly reduced the expression of A20 in rotavirus-infected PIE cells (15), which was in line with the capacity of both strains to improve IRF3 activation and IFN-β production. In line with our findings, MacPherson et al. (23) showed that the stimulation of HT29 cells with poly(I:C) alone increased the expression of A20, but the co-stimulation with poly(I:C) and probiotics significantly reduced A20 expression levels. Although our microarray analysis did not show differences between lactobacilli-treated and control PIE cells when the *A20* (*TNFAIP3*) transcript was evaluated, qPCR analysis showed a significant reduction of A20 expression in immunobiotic-treated cells. Therefore, the reduction of A20 in IECs could be a key effect for the antiviral capabilities of immunobiotics.

*Lactobacillus rhamnosus* CRL1505 and *L. plantarum* CRL1506 showed quantitative and qualitative differences in their capacities to modulate the innate antiviral immune response in PIE cells. Higher expression levels of the antiviral factors *MX2* and *IFIT2* were found in CRL1505-treated PIE cells when compared to CRL1506-treated cells. Moreover, some antiviral factors were upregulated only with *L. rhamnosus* CRL1505 treatment including *IFIT1, IFIT3, RIG-1, MDA5, NLRP3*, and *MSX1*. As mentioned before, *RIG-1, MDA5*, and *NLRP3* are important factors in the protection against gastrointestinal virus such as rotavirus. In addition, *MSX1* (also known as *HOX7*) was recently identified as an important modulator of RIG-1-mediated signaling pathway with the ability to induce the activation of the TBK1 kinase and IRF3, increasing the expression of antiviral genes and improving innate antiviral responses (40). Furthermore, *L. rhamnosus* CRL1505 differentially regulated the expression of proinflamamtory and anti-inflammatory factors in poly(I:C)-challenged PIE cells. Higher expression of *CCL4, CCL5, CCL20*, and *CXCL10* were found in CRL1505-treated PIE cells when compared to CRL1506-treated cells, whereas *CXCL2, CXCL5*, and *CXCL11* were upregulated only with *L. rhamnosus* CRL1505 treatment, indicating a higher capacity of this strain to induce recruitment of immune cells. It also seems that the CRL1505 strain would have a higher ability to improve the regulation of the inflammatory response. We observed higher expression of *PLA2G4A* and *PTGES* that would enhance the production of the anti-inflammatory PGE2. Of interest, microarray analysis showed an increase in the expression of IL-27 in *L. rhamnosus* CRL1505 treatment, an effect that was not observed in the other experimental groups. IL-27 is a member of IL-12 family of cytokines that is produced mainly by myeloid cell populations, including macrophages, inflammatory monocytes, and dendritic cells, but its production has been reported in endothelial cells and epithelial cells as well (41). This cytokine has important roles in the early regulation of Th1 differentiation and the suppression of cellular activation and production of proinflammatory cytokines (42). It was demonstrated that IL-27 induces IL-10 production from both mouse and human CD4^+^ and CD8^+^ T cells and NK cells (43). Moreover, some recent studies reported a role for this cytokine in restricting virus replication (42). These effects would explain the higher capacity of the CRL1505 strain when compared to CRL1506 to protect against viral infection and inflammatory damage (5, 11, 19).

In conclusion, the genome-wide transcriptional profiling performed in this work allowed us to obtain a global overview of the expression patterns of immune and immune-related genes involved in the response of PIE cells to poly(I:C) stimulation. This study also confirmed that *L. rhamnosus* CRL1505 and *L. plantarum* CRL1506 differently modulate gene expression in poly(I:C)-challenged PIE cells inducing changes that could help to explain the antiviral activities observed in animal models and clinical trials. These results provided clues for the better understanding the mechanism underlying host–immunobiotic interaction.

The main outcome from the study is that our transcriptomic analysis successfully identified a group of genes (*IFN-*β, *RIG1, RNASEL, MX2, A20, IL27, CXCL5, CCL4, PTGES*, and *PTGER4*), which can be used as prospective biomarkers for the screening of new antiviral immunobiotics in PIE cells. Classically, the selection of potential immunobiotic strains is performed by studying few biomarkers *in vitro*, and in many cases, the selected strains do not exhibit the same immunomodulatory activity when they are evaluated later in *in vivo* models. Our preliminary studies indicate that the set of biomarkers found in this work allows an efficient *in vitro* selection of new strains with antiviral activity in PIE cells, which present antiviral activity when they are evaluated later in animal models. This efficient selection of immunobiotics could improve the development of novel functional food and feeds, which may help to prevent viral infections.

## Author Contributions

HA, SA, JV, and HaK designed the study and manuscript writing. LA, HisK, HI, and NS did the laboratory work in the expression and statistical analysis. LA and JV participated in the data analysis of microarray. SS, TN, JV, and HaK contributed to data analysis and interpretation. All the authors read and approved the manuscript.

## Conflict of Interest Statement

The authors declare that the research was conducted in the absence of any commercial or financial relationships that could be construed as a potential conflict of interest.

## Supplementary Material

Figure S1 **Expression of adhesion molecules genes in porcine intestinal epithelial (PIE) cells after the challenge with the viral molecular associated pattern poly(I:C), analyzed by quantitative PCR**. The results represent data from three independent experiments. Symbols indicate significant differences when compared to unchallenged control PIE cells (time 0 h) (**P* < 0.05, ***P* < 0.01).

Figure S2 **Expression of trefoil factor 1, lysozyme, granzyme, and A20 genes in porcine intestinal epithelial (PIE) cells after the challenge with the viral molecular associated pattern poly(I:C), analyzed by quantitative PCR**. The results represent data from three independent experiments. Symbols indicate significant differences when compared to unchallenged control PIE cells (time 0 h) (**P* < 0.05, ***P* < 0.01, ^#^*P* < 0.001).

Figure S3 **Heat map analysis of the differentially regulated genes in porcine intestinal epithelial (PIE) cells treated with immunobiotic *Lactobacillus rhamnosus* CRL1505 or *Lactobacillus plantarum* CRL1506 and challenged with the viral molecular associated pattern poly(I:C)**. Non-lactobacilli-treated PIE cells challenged with poly(I:C) were used as controls.

Figure S4 **Expression of trefoil factor 1, lysozyme, granzyme, serum amyloid A2, and complement system factors genes in porcine intestinal epithelial (PIE) cells treated with immunobiotic *Lactobacillus rhamnosus* CRL1505 or *Lactobacillus plantarum* CRL1506 and challenged with the viral molecular associated pattern poly(I:C), analyzed by quantitative PCR**. Non-lactobacilli-treated PIE cells with or without poly(I:C) challenge were used as controls. The results represent data from three independent experiments. Asterisks indicate significant differences when compared to poly(I:C)-challenged control PIE cells (**P* < 0.05).
