# Supplementary material for: Transcriptomic Analysis of the Innate Antiviral Immune Response in Porcine Intestinal Epithelial Cells: Influence of Immunobiotic Lactobacilli
Source: Front Immunol. 2017 Feb 2;8:57. doi: 10.3389/fimmu.2017.00057 (PMC5288346; doi:10.3389/fimmu.2017.00057)
Supplement: Supplementary file 1 [file Table_1.DOCX]

**Supplemental Table 1:** Transcripts modulated by Poly(I:C) challenge in porcine intestinal epithelial (PIE) cells. Microarray data expressed as a log_2_ fold-change of Poly(I:C) stimulated-PIE cells versus unchallenged control PIE cells at hour 12. Immune gene function data are from GeneCard. N.M.: not modified. Significantly different with respect to poly(I:C) control * p<0.05, **p<0.01.

**Interferons, interferon-induced proteins and antiviral factors**

| **Probe Name** | **Gene Symbol** | **Poly(I:C)** | **CRL1505 + Poly(I:C)** | **CRL1506 + Poly(I:C)** | **Gene Name** |
| --- | --- | --- | --- | --- | --- |
| A_72_P303019 | RSAD2 | 13.437 | 13.862 | 13.613 | radical S-adenosyl methionine domain-containing protein 2 |
| A_72_P223657 | OAS1 | 11.249 | 12.795* | 12.495* | 2'-5'-oligoadenylate synthetase 1 |
| A_72_P177731 | OASL | 10.790 | 11.447* | 11.142* | 2'-5'-oligoadenylate synthetase-like |
| A_72_P489826 | IFIT1 | 9.906 | 10.462* | 10.104 | interferon-induced protein with tetratricopeptide repeats 1 |
| A_72_P489821 | IFIT3 | 9.106 | 9.724* | 9.427 | interferon-induced protein with tetratricopeptide repeats 3 |
| A_72_P223287 | DDX58 | 8.936 | 9.488* | 9.273 | dead box polypeptide 58, also known as RIG-I |
| A_72_P002311 | IFIT2 | 8.304 | 9.284** | 8.689* | interferon-induced protein with tetratricopeptide repeats 2 |
| A_72_P010326 | MX1 | 7.978 | 8.043 | 8.011 | myxovirus resistance 1, interferon-inducible protein p78 |
| A_72_P191026 | HERC5 | 6.621 | 6.811 | 6.713 | HECT and RLD domain containing E3 ubiquitin protein ligase 5 |
| A_72_P444275 | OAS2 | 6.398 | 6.706 | 6.513 | 2'-5'-oligoadenylate synthetase 2 |
| A_72_P177406 | MX2 | 6.305 | 6.956* | 6.638* | myxovirus resistance 2 |
| A_72_P172311 | IFITM1 | 5.763 | 6.329* | 6.005 | interferon induced transmembrane protein 1 |
| A_72_P213717 | DHX58 | 5.659 | 6.147* | 5.941 | dexh box polypeptide 58 |
| A_72_P066761 | IFIH1 | 5.401 | 6.080* | 5.638 | interferon induced with helicase C domain 1 |
| A_72_P441219 | ISG15 | 4.949 | 5.036 | 4.997 | ISG15 ubiquitin-like modifier |
| A_72_P207667 | IFI44 | 4.664 | 4.808 | 4.810 | interferon-induced protein 44 |
| A_72_P077861 | IRF7 | 4.637 | 5.093* | 4.840 | interferon regulatory factor 7 |
| A_72_P703420 | STAT1 | 4.388 | 4.956* | 4.698 | signal transducer and activator of transcription 1 |
| A_72_P223342 | IFNB1 | 4.326 | 5.667** | 4.732* | interferon beta 1 |
| A_72_P002976 | NLRP3 | 3.984 | 4.455* | 4.091 | NLR family pyrin domain containing 3 |
| A_72_P536281 | IRF1 | 3.972 | 4.595* | 4.172 | interferon regulatory factor 1 |
| A_72_P081716 | IFI35 | 3.616 | 3.991 | 3.810 | interferon-induced protein 35 |
| A_72_P077906 | IFNA1 | 3.562 | 4.876** | 4.234* | interferon alpha 1 |
| A_72_P441748 | PKR | 3.257 | 3.492 | 3.377 | double stranded RNA-dependent protein kinase |
| A_72_P667411 | IFI30 | 3.136 | 3.515* | 3.319 | interferon gamma-inducible protein 30 |
| A_72_P489831 | IFIT5 | 3.000 | 3.381 | 3.174 | interferon-induced protein with tetratricopeptide repeats 5 |
| A_72_P097261 | MB21D1 | 2.810 | 3.385* | 3.036 | Mab-21 domain containing 1 |
| A_72_P223322 | IRF9 | 2.547 | 2.614 | 2.527 | interferon regulatory factor 9 |
| A_72_P775183 | TRIM21 | 2.440 | 3.052* | 2.644 | tripartite motif containing 21 |
| A_72_P088106 | STAT2 | 2.243 | 2.873* | 2.538 | signal transducer and activator of transcription 2 |
| A_72_P211817 | RNASEL | 2.219 | 2.963* | 2.704* | ribonuclease L (2',5'-oligoisoadenylate synthetase-dependent) |
| A_72_P302994 | RNASE4 | 1.986 | 2.661* | 2.244* | ribonuclease RNase A family 4 |
| A_72_P062151 | MSX1 | 1.516 | 2.102* | 1.782 | msh homeobox 1 |
| A_72_P443262 | IRF2 | 1.030 | 1.581* | 1.235 | interferon regulatory factor 2 |
| A_72_P232807 | STAT3 | -1.120 | N.M. | N.M. | signal transducer and activator of transcription |
| A_72_P242752 | RNASE6 | -2.074 | -1.587 | N.M. | ribonuclease RNase A family k6 |
| A_72_P284859 | ATF4 | N.M. | 1.323* | 1.164* | activating transcription factor 4 |
| A_72_P165194 | IRF3 | N.M. | 1.088* | 1.025* | interferon regulatory factor 3 |
| A_72_P036011 | STAT5A | N.M. | 1.956* | 1.049* | signal transducer and activator of transcription 5A |
|  |  |  |  |  |  |

**Cytokine and chemokine signaling pathways**

| **Probe Name** | **Gene Symbol** | **Poly(I:C)** | **CRL1505 + Poly(I:C)** | **CRL1506 + Poly(I:C)** | **Gene Name** |
| --- | --- | --- | --- | --- | --- |
| A_72_P035541 | CASP1 | 4.887 | 5.886** | 5.412* | caspase 1, apoptosis-related cysteine peptidase |
| A_72_P418159 | TNFSF10 | 4.222 | 5.065* | 4.455 | tumor necrosis factor (ligand) superfamily, member 10 |
| A_72_P444799 | IL1A | 4.187 | 4.825* | 4.564* | interleukin 1 alpha |
| A_72_P680394 | IL6 | 4.074 | 5.235* | 4.785* | interleukin 6 |
| A_72_P374863 | TNFAIP3 | 3.656 | 3.846 | 3.485 | tumor necrosis factor alpha-induced protein 3, also known as A20 |
| A_72_P410733 | BTC | 3.522 | 3.907 | 3.776 | betacellulin |
| A_72_P032536 | TNFAIP6 | 2.829 | 4.079* | 3.940* | tumor necrosis factor, alpha-induced protein 6 |
| A_72_P302389 | IL20RB | 2.316 | N.M.* | -1.091* | interleukin 20 receptor beta |
| A_72_P035856 | IL15 | 1.837 | 1.124* | 1.395* | interleukin 15 |
| A_72_P108391 | TGFBR3 | 1.714 | 2.646* | 2.256* | transforming growth factor beta receptor III |
| A_72_P441494 | VEGFA | 1.683 | 2.140* | 1.905 | vascular endothelial growth factor A |
| A_72_P250642 | CASP4 | 1.602 | 2.376* | 1.976 | caspase 4, apoptosis-related cysteine peptidase |
| A_72_P146321 | EGF | 1.475 | 1.270 | 1.860* | epidermal growth factor |
| A_72_P466671 | IL13RA2 | 1.458 | 1.646 | 1.734 | interleukin 13 receptor alpha 2 |
| A_72_P559234 | TNIP1 | 1.297 | 1.581 | 1.424 | TNFAIP3 interacting protein 1 |
| A_72_P674666 | TNFSF13B | 1.274 | 2.990* | 3.028* | tumor necrosis factor (ligand) superfamily, member 13b |
| A_72_P413808 | IL8 (CXCL8) | 1.225 | 1.876* | 1.702* | interleukin 8 |
| A_72_P078026 | SOCS2 | -1.026 | -1.653* | -1.388 | suppressor of cytokine signaling 2 |
| A_72_P541206 | TGFB2 | -1.307 | -1.508 | -1.505 | transforming growth factor beta 2 |
| A_72_P075046 | IL9 | -2.036 | -3.167* | -1.833 | interleukin 9 |
| A_72_P077441 | TNFSF4 | -2.376 | -1.516 | -2.937 | tumor necrosis factor (ligand) superfamily, member 4 |
| A_72_P440361 | IL23RA | -2.611 | N.M.* | -2.203 | interleukin 23 receptor alpha |
| A_72_P302989 | ADAM17 | N.M. | 1.359* | 1.199* | ADAM metallopeptidase domain 17 |
| A_72_P440121 | AREG | N.M. | 1.238* | 1.078* | amphiregulin |
| A_72_P267601 | IL17RC | N.M. | 1.124* | N.M. | interleukin 17 receptor C |
| A_72_P466532 | IL1RAPL2 | N.M. | N.M. | 1.540* | interleukin 1 receptor accessory protein-like 2 |
| A_72_P165821 | IL27 | N.M. | 1.077* | N.M. | interleukin 27 |
| A_72_P475097 | TNFAIP8L2 | N.M. | N.M. | 2.043* | tumor necrosis factor, alpha-induced protein 8-like 2 |
| A_72_P473423 | TNFRSF11B | N.M. | -1.261* | N.M. | tumor necrosis factor receptor superfamily, member 11b |
| A_72_P443210 | TNFSF18 | N.M. | N.M. | 4.070* | tumor necrosis factor (ligand) superfamily, member 18 |
| A_72_P718908 | TRAF5 | N.M. | -1.076* | -1.032* | TNF receptor-associated factor 5 |
| A_72_P165266 | CXCL10 | 13.232 | 14.187** | 13.768* | chemokine (C-X-C motif) ligand 10 |
| A_72_P442072 | CXCL11 | 10.365 | 11.024* | 10.575 | chemokine (C-X-C motif) ligand 11 |
| A_72_P372628 | CCL5 | 8.421 | 9.461** | 8.968* | chemokine (C-C motif) ligand 5 |
| A_72_P442032 | CXCL9 | 8.186 | 8.185 | 7.770 | chemokine (C-X-C motif) ligand 9 |
| A_72_P223332 | CCL4 | 7.844 | 8.621** | 8.082* | chemokine (C-C motif) ligand 4 |
| A_72_P177441 | CCL20 | 5.995 | 7.080** | 6.323* | chemokine (C-C motif) ligand 20 |
| A_72_P326028 | CCL23 | 5.195 | 5.835* | 5.796* | chemokine (C-C motif) ligand 23 |
| A_72_P442316 | CCL11 | 3.918 | 3.805 | 4.175 | chemokine (C-C motif) ligand 11 |
| A_72_P077726 | CSF2 | 2.996 | 3.551* | 3.367* | colony stimulating factor 2 (granulocyte-macrophage) |
| A_72_P077321 | CCL28 | 2.945 | 3.988* | N.M.* | chemokine (C-C motif) ligand 28 |
| A_72_P175796 | CSF1 | 2.943 | 3.148 | 2.923 | colony stimulating factor 1 (macrophage) |
| A_72_P146431 | CCRL2 | 2.843 | 3.304* | 2.928 | chemokine (C-C motif) receptor-like 2 |
| A_72_P035338 | CXCL5 | 2.500 | 3.308* | 2.652 | alveolar macrophage-derived chemotactic factor-II |
| A_72_P732383 | CXCL2 | 2.477 | 3.382* | 2.698 | chemokine (C-X-C motif) ligand 2 |
| A_72_P441594 | CCL8 | 2.355 | 2.590 | 2.413 | chemokine (C-C motif) ligand 8 |
| A_72_P675364 | CCL2 | 2.014 | 2.262 | 2.181 | chemokine (C-C motif) ligand 2 |
| A_72_P035356 | CCL3L1 | 1.507 | 2.900* | 2.723* | chemokine (C-C motif) ligand 3-like 1 |
| A_72_P146516 | CCR5 | 1.356 | 1.414 | 1.168 | chemokine (C-C motif) receptor 5 |
| A_72_P074981 | CX3CL1 | 1.169 | 1.429 | 1.060 | chemokine (C-X3-C motif) ligand 1 |
| A_72_P713558 | CXCL12 | -1.154 | -1.232 | -1.173 | chemokine (C-X-C motif) ligand 12 |
| A_72_P210682 | CXCL14 | -1.413 | -1.959 | -1.633 | chemokine (C-X-C motif) ligand 14 |
| A_72_P223792 | CCR7 | N.M. | N.M. | 1.448* | chemokine (C-C motif) receptor 7 |
| A_72_P428729 | S100A8 | N.M. | -2.530* | -1.234* | S100 calcium binding protein A8 |
|  |  |  |  |  |  |

**Adhesion Molecules**

| **Probe Name** | **Gene Symbol** | **Poly(I:C)** | **CRL1505 + Poly(I:C)** | **CRL1506 + Poly(I:C)** | **Gene Name** |
| --- | --- | --- | --- | --- | --- |
| A_72_P440571 | LGALS9 | 7.131 | 7.360 | 7.234 | lectin, galactoside-binding, soluble, 9 |
| A_72_P035596 | SELE | 5.358 | 6.062* | 5.476 | selectin E |
| A_72_P052836 | CDHR4 | 4.055 | 5.043* | 4.455 | cadherin-related family member 4 |
| A_72_P088576 | VCAM1 | 4.007 | 4.326 | 4.097 | vascular cell adhesion molecule 1 |
| A_72_P372738 | SELL | 2.638 | 3.359** | 3.093* | selectin L |
| A_72_P223937 | ICAM-1 | 2.121 | 2.826* | 2.477 | intercellular adhesion molecule-1 |
| A_72_P088181 | VTN | 2.107 | -1.950* | N.M.* | vitronectin |
| A_72_P075056 | RAE1 | 2.952 | 1.633* | 1.784* | retinoic acid early inducible-1 |
| A_72_P729903 | EPCAM | 1.882 | 3.651* | 1.981 | epithelial cell adhesion molecule |
| A_72_P232527 | SELP | 1.841 | 2.037 | 1.868 | selectin P |
| A_72_P146616 | ITGB2 | 1.076 | 1.549 | 1.426 | integrin beta 2 |
| A_72_P028406 | ITGA5 | -1.015 | -1.139 | -1.216 | integrin alpha 5 |
| A_72_P479115 | FLRT2 | -1.054 | -1.385 | -1.280 | fibronectin leucine rich transmembrane protein 2 |
| A_72_P366488 | ITGA1 | -1.260 | N.M.* | -1.033 | integrin alpha 1 |
| A_72_P496491 | THBS1 | -1.373 | -1.220 | -1.350 | thrombospondin 1 |
| A_72_P527727 | ITGB5 | -2.284 | -2.365 | -2.077 | integrin beta 5 |
| A_72_P308833 | CADM4 | N.M. | N.M. | -1.013* | cell adhesion molecule 4 |
| A_72_P493499 | CDH19 | N.M. | N.M. | 2.830* | cadherin 19 type 2 |
| A_72_P478143 | CDH24 | N.M. | N.M. | 1.231* | cadherin 24 type 2 |
| A_72_P176186 | IBSP | N.M. | 4.328* | N.M. | integrin-binding sialoprotein |
|  |  |  |  |  |  |

**Prostaglandins**

| **Probe Name** | **Gene Symbol** | **Poly(I:C)** | **CRL1505 + Poly(I:C)** | **CRL1506 + Poly(I:C)** | **Gene Name** |
| --- | --- | --- | --- | --- | --- |
| A_72_P302784 | PTGS2 | 5.083 | 6.787** | 6.389* | prostaglandin-endoperoxide synthase 2 (prostaglandin G/H synthase) |
| A_72_P230127 | PTGIR | 3.632 | 4.373* | 3.900 | prostaglandin I2 (prostacyclin) receptor (IP) |
| A_72_P082476 | PTGIS | 1.665 | 1.771 | 1.543 | prostaglandin I2 (prostacyclin) synthase |
| A_72_P101606 | PTGER4 | 1.640 | -1.471* | -1.242* | prostaglandin E receptor 4 (subtype EP4) |
| A_72_P006091 | PLA2G4A | 1.281 | 1.820* | 1.693* | phospholipase A2, group IVA (cytosolic, calcium-dependent) |
| A_72_P153186 | ALOX5AP | -1.115 | -1.243 | -1.084 | arachidonate 5-lipoxygenase-activating protein |
| A_72_P035646 | DPEP1 | N.M. | N.M. | 2.728* | renal dipeptidase 1 |
| A_72_P652275 | PTGER2 | N.M. | -1.184* | N.M. | prostaglandin E receptor 2 subtype EP2 |
| A_72_P177991 | PTGES | N.M. | 1.164* | 1.512* | prostaglandin E synthase |
| A_72_P235687 | PTGFRN | N.M. | -1.999* | -2.570* | prostaglandin F2 receptor negative regulator |
|  |  |  |  |  |  |

**Pattern Recognition Receptors and Signaling**

| **Probe Name** | **Gene Symbol** | **Poly(I:C)** | **CRL1505 + Poly(I:C)** | **CRL1506 + Poly(I:C)** | **Gene Name** |
| --- | --- | --- | --- | --- | --- |
| A_72_P088191 | PGLYRP2 | 8.179 | 7.950 | 7.607 | peptidoglycan recognition protein 2 |
| A_72_P032641 | IRG1 | 5.119 | 6.353* | 5.831* | immunoresponsive 1 homolog |
| A_72_P471206 | TLR3 | 2.843 | 3.400* | 3.139* | toll-like receptor 3 |
| A_72_P342398 | NFKBIA | 2.653 | 3.216* | 2.841 | nuclear factor of kappa inhibitor alpha |
| A_72_P088356 | TLR2 | 1.678 | 1.835 | 1.791 | toll-like receptor 2 |
| A_72_P428259 | TRAF3IP2 | 1.329 | 1.858* | 1.327 | TRAF3 interacting protein 2 |
| A_72_P488289 | TRAFD1 | 1.038 | 1.495* | 1.296 | TRAF-type zinc finger domain containing 1 |
| A_72_P441798 | PPARA | -1.759 | N.M.* | N.M.* | peroxisome proliferator-activated receptor alpha |
| A_72_P077836 | PPARGC | -2.279 | -2.316 | -2.300 | peroxisome proliferator activated receptor gamma, coactivator 1 alpha |
| A_72_P088166 | TLR6 | N.M. | 1.815* | 1.449* | toll-like receptor 6 |
| A_72_P245972 | MYD88 | N.M. | 1.140* | 1.004* | myeloid differentiation primary response gene (88) |
| A_72_P020616 | NCOA1 | N.M. | 1.564* | 1.105* | nuclear receptor coactivator 1 |
| A_72_P146316 | NFKB1 | N.M. | 1.179* | 1.015* | nuclear factor of kappa light polypeptide gene enhancer in B-cells 1 |
| A_72_P036221 | NFIA | N.M. | N.M. | -1.351* | nuclear factor I/A |
| A_72_P516012 | NFIB | N.M. | -1.036* | -1.073* | nuclear factor I/B |
| A_72_P232232 | PIK3R5 | N.M. | 1.055* | N.M. | phosphoinositide-3-kinase, regulatory subunit 5 |
|  |  |  |  |  |  |

**Other immune and immune-related genes**

| **Probe Name** | **Gene Symbol** | **Poly(I:C)** | **CRL1505 + Poly(I:C)** | **CRL1506 + Poly(I:C)** | **Gene Name** |
| --- | --- | --- | --- | --- | --- |
| A_72_P511075 | CHI3L1 | 8.413 | 9.069* | 8.891 | chitinase 3-like 1 |
| A_72_P539698 | DUOX1 | 2.426 | N.M.* | N.M.* | dual oxidase 1 |
| A_72_P478010 | GZMH | 1.014 | 2.895** | 1.651* | granzyme H |
| A_72_P172986 | TFF1 | -1.499 | N.M.* | N.M.* | trefoil factor 1 |
| A_72_P213667 | BPI | -3.563 | -3.598 | -2.519* | bactericidal/permeability-increasing protein |
| A_72_P540518 | BPIFA1 | N.M. | N.M. | 3.159* | BPI fold containing family A, member 1 |
| A_72_P177571 | LYZ | N.M. | 2.533* | 1.937* | Lysozyme |
| A_72_P345253 | C1R | 7.533 | 8.390* | 7.959* | complement component 1, r subcomponent |
| A_72_P088781 | C1S | 5.713 | 6.672* | 6.287 | complement component 1, s subcomponent |
| A_72_P164781 | C5AR1 | 5.290 | 5.777 | 5.938 | complement component 5a receptor 1 |
| A_72_P302604 | CFB | 3.936 | 1.699* | 1.663* | complement factor B |
| A_72_P088401 | PLG | 3.452 | 1.118* | N.M.* | Plasminogen |
| A_72_P742730 | C3 | 2.948 | 3.816* | 3.141 | complement component 3 |
| A_72_P010246 | PLAU | 2.921 | 3.655* | 3.291 | plasminogen activator, urokinase |
| A_72_P232632 | PROC | 1.952 | 1.970 | N.M.* | protein C (inactivator of coagulation factors Va and VIIIa) |
| A_72_P039081 | CFD | 1.648 | 2.066* | 1.998* | complement factor D |
| A_72_P706393 | MASP1 | -1.065 | -1.621* | -1.111 | mannan-binding lectin serine peptidase 1 |
| A_72_P029441 | TFPI | -1.229 | -1.754 | -1.614 | tissue factor pathway inhibitor |
| A_72_P293294 | VWF | -2.382 | -2.871 | -2.758 | von Willebrand factor |
| A_72_P467035 | F9 | -2.536 | -2.606 | -2.377 | coagulation factor IX |
| A_72_P686307 | C5 | N.M. | -1.202* | N.M. | complement component 5 |
| A_72_P766152 | SAA2 | 8.641 | 9.574* | 9.299* | serum amyloid A2 |
| A_72_P669424 | SOD2 | 3.593 | 3.844 | 3.731 | superoxide dismutase 2, mitochondrial |
| A_72_P383948 | NRROS | -1.089 | -1.308 | -1.186 | negative regulator of reactive oxygen species |
| A_72_P441199 | NOS2 | N.M. | 1.934* | 1.773* | nitric oxide synthase 2 inducible |
| A_72_P082446 | SEPN1 | N.M. | -1.075* | -1.021* | selenoprotein N 1 |
|  |  |  |  |  |  |
|  |  |  |  |  |  |
