# Supplementary figures and images for: Transcriptomic Analysis of the Innate Antiviral Immune Response in Porcine Intestinal Epithelial Cells: Influence of Immunobiotic Lactobacilli
Source: Front Immunol. 2017 Feb 2;8:57. doi: 10.3389/fimmu.2017.00057 (PMC5288346; doi:10.3389/fimmu.2017.00057)

## EPCAM

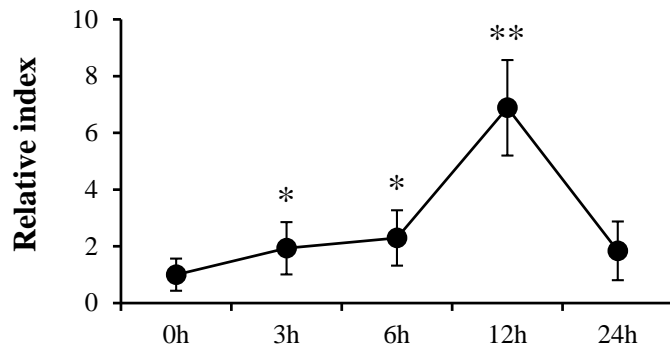

## ICAM1

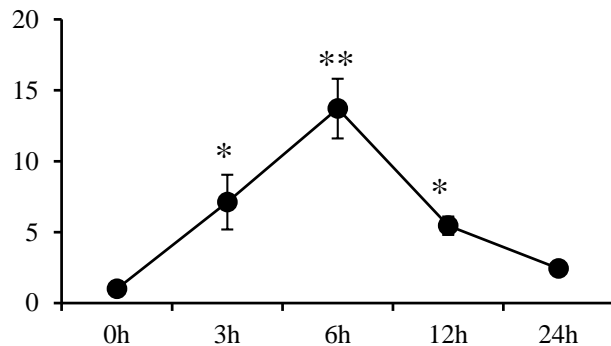

## SELE

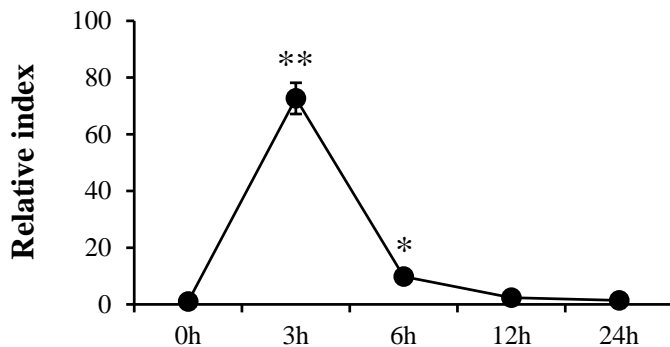

## SELL

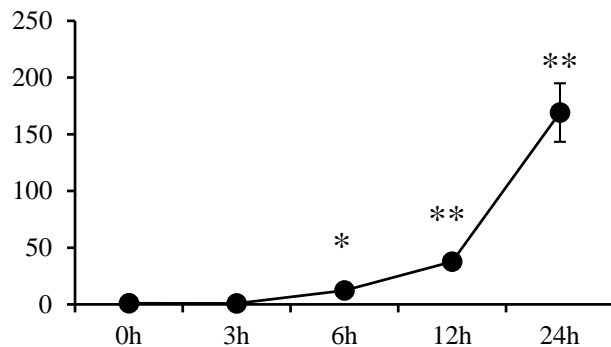

Supplement: Figure S1 — Expression of adhesion molecules genes in porcine intestinal epithelial (PIE) cells after the challenge with the viral molecular associated pattern poly(I:C), analyzed by quantitative PCR. The results represent data from three independent experiments. Symbols indicate significant differences when compared to unchallenged control PIE cells (time 0 h) (*P < 0.05, **P < 0.01). [file Image_1.PDF]

**TFF-1**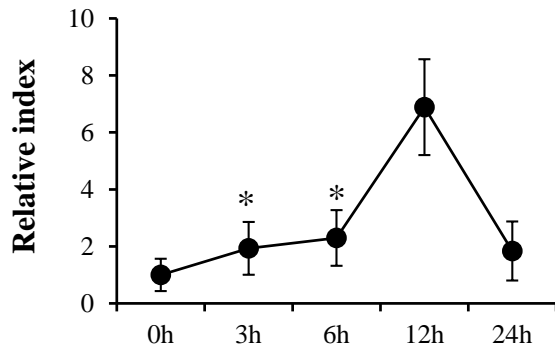**LYZ**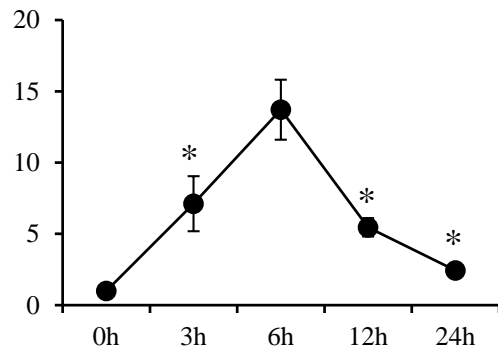**GZMA**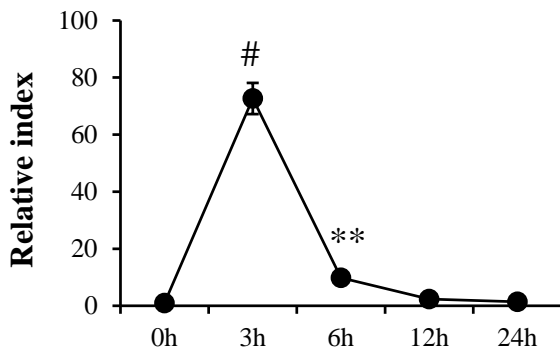**SAA2**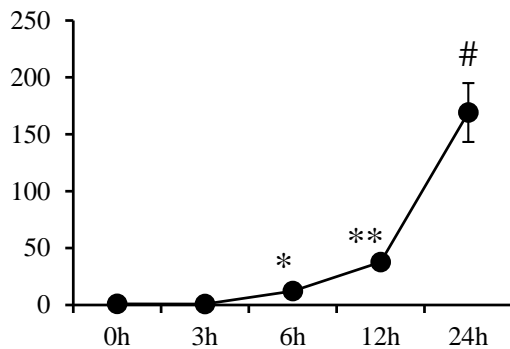

Supplement: Figure S2 — Expression of trefoil factor 1, lysozyme, granzyme, and A20 genes in porcine intestinal epithelial (PIE) cells after the challenge with the viral molecular associated pattern poly(I:C), analyzed by quantitative PCR. The results represent data from three independent experiments. Symbols indicate significant differences when compared to unchallenged control PIE cells (time 0 h) (*P < 0.05, **P < 0.01, #P < 0.001). [file Image_2.PDF]

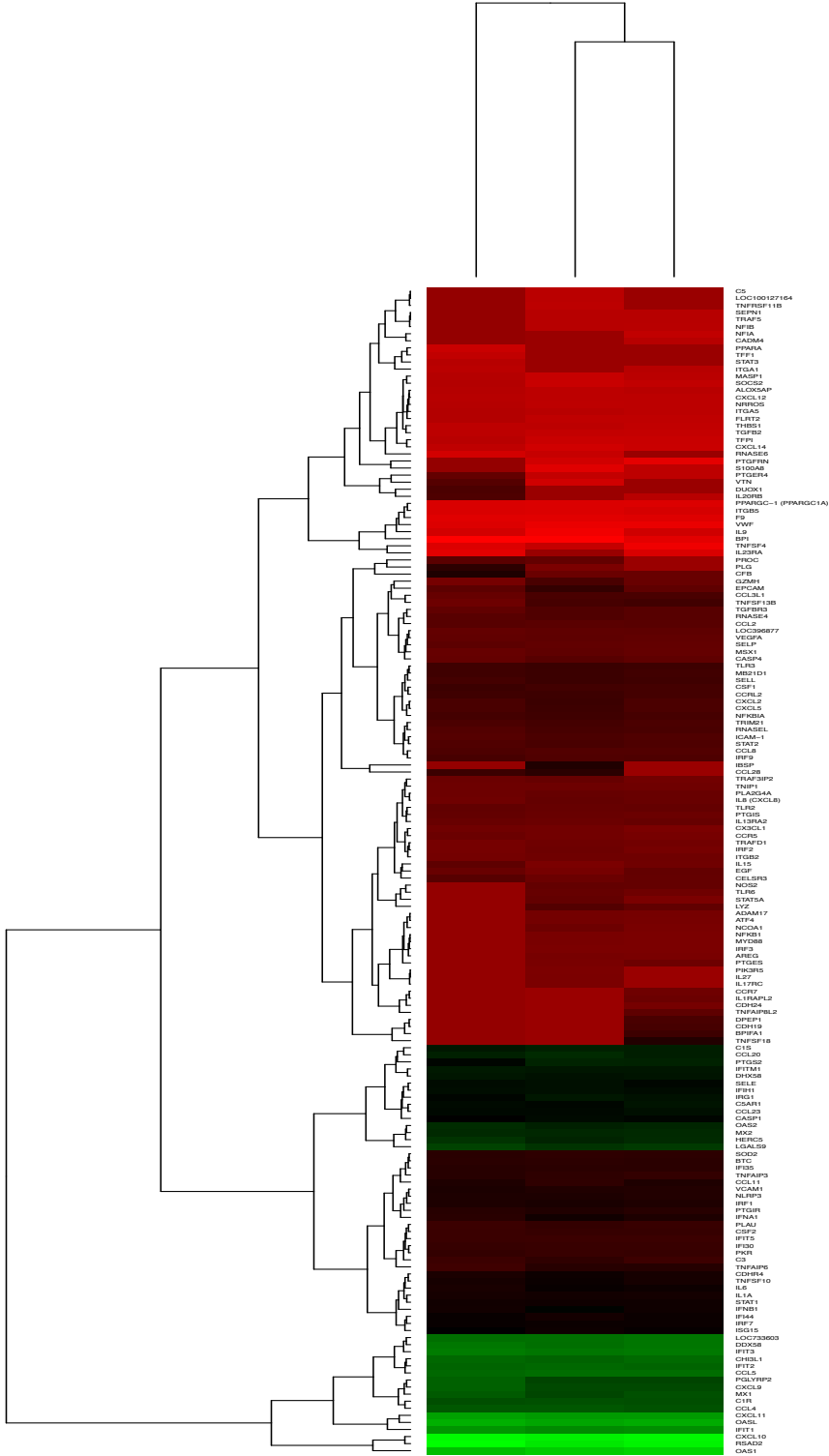

Poly(I:C)

CRL1505  
+ Poly(I:C)

CRL1506  
+ Poly(I:C)

Supplement: Figure S3 — Heat map analysis of the differentially regulated genes in porcine intestinal epithelial (PIE) cells treated with immunobiotic Lactobacillus rhamnosus CRL1505 or Lactobacillus plantarum CRL1506 and challenged with the viral molecular associated pattern poly(I:C). Non-lactobacilli-treated PIE cells challenged with poly(I:C) were used as controls. [file Image_3.PDF]

**TFF1**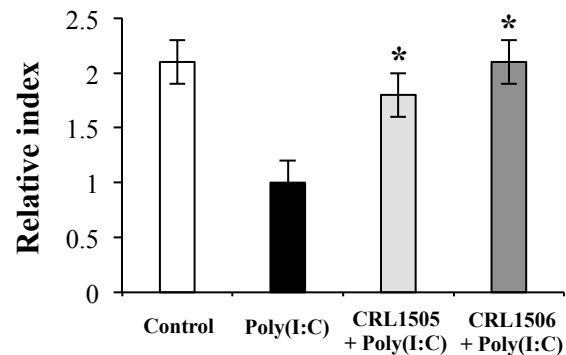**LYZ**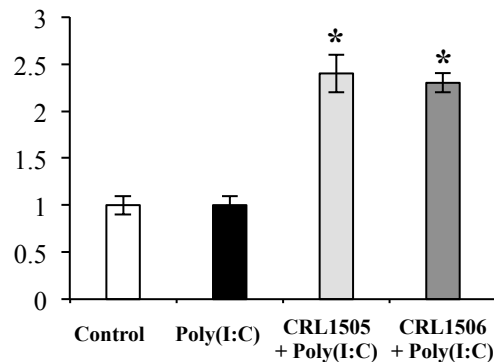**GZMA**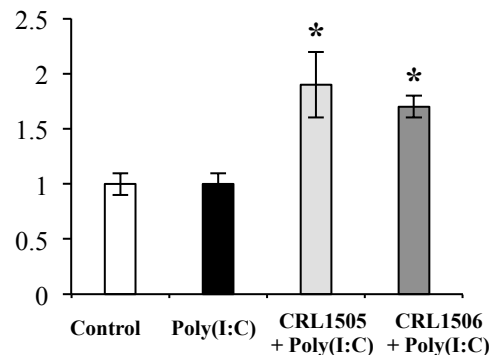**SAA2**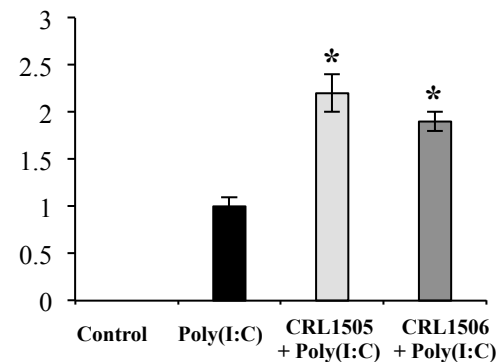**C1R**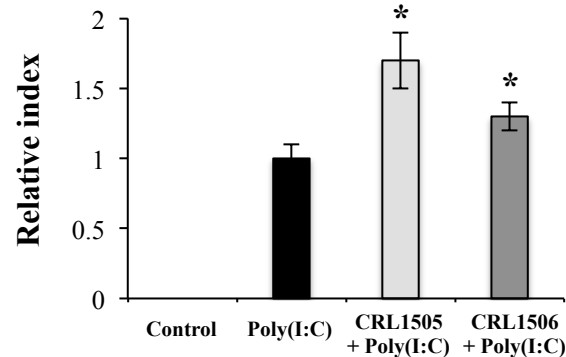**C1S**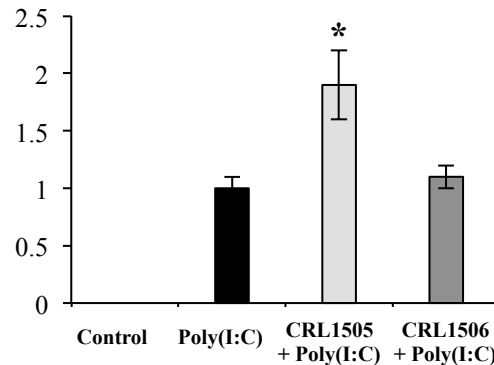**C3**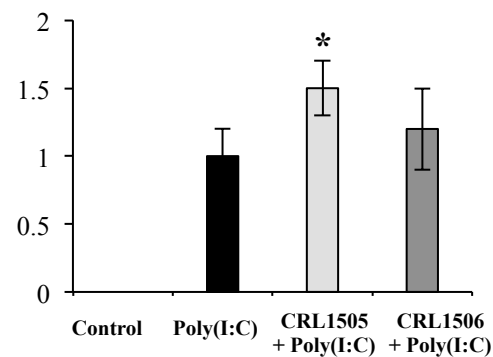**CFB**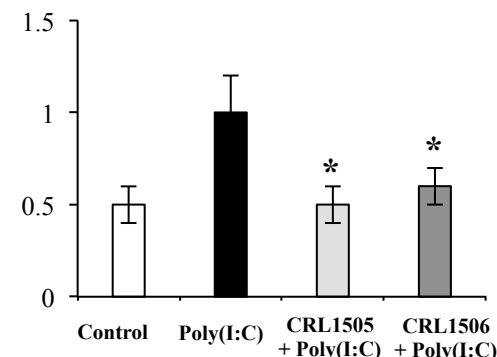

Supplement: Figure S4 — Expression of trefoil factor 1, lysozyme, granzyme, serum amyloid A2, and complement system factors genes in porcine intestinal epithelial (PIE) cells treated with immunobiotic Lactobacillus rhamnosus CRL1505 or Lactobacillus plantarum CRL1506 and challenged with the viral molecular associated pattern poly(I:C), analyzed by quantitative PCR. Non-lactobacilli-treated PIE cells with or without poly(I:C) challenge were used as controls. The results represent data from three independent experiments. Asterisks indicate significant differences when compared to poly(I:C)-challenged control PIE cells (*P < 0.05). [file Image_4.PDF]
